# Preserved and variable spatial‐chemical changes of lipids across tomato leaves in response to central vein wounding reveals potential origin of linolenic acid in signal transduction cascade

**DOI:** 10.1002/pei3.10038

**Published:** 2021-02-01

**Authors:** Dušan Veličković, Rosalie K. Chu, Corinna Henkel, Annika Nyhuis, Nannan Tao, Jennifer E. Kyle, Joshua N. Adkins, Christopher R. Anderton, Vanessa Paurus, Kent Bloodsworth, Lisa M. Bramer, Dale S. Cornett, Wayne R. Curtis, Kristin E. Burnum‐Johnson

**Affiliations:** ^1^ Environmental Molecular Sciences Laboratory Pacific Northwest National Laboratory Richland WA USA; ^2^ Bruker Daltonik GmbH Bremen Germany; ^3^ Bruker Daltonics San Jose CA USA; ^4^ Biological Sciences Division Pacific Northwest National Laboratory Richland WA USA; ^5^ Computing & Analytics Division Pacific Northwest National Laboratory Richland WA USA; ^6^ Bruker Daltonics Billerica MA USA; ^7^ Department of Chemical Engineering The Pennsylvania State University University Park PA USA

**Keywords:** lysophospholipid, lysophosphatidylcholine, MALDI, MS imaging, spatial lipidome, environmental stress, wounding

## Abstract

Membrane lipids serve as substrates for the generation of numerous signaling lipids when plants are exposed to environmental stresses, and jasmonic acid, an oxidized product of 18‐carbon unsaturated fatty acids (e.g., linolenic acid), has been recognized as the essential signal in wound‐induced gene expression. Yet, the contribution of individual membrane lipids in linolenic acid generation is ill‐defined. In this work, we performed spatial lipidomic experiments to track lipid changes that occur locally at the sight of leaf injury to better understand the potential origin of linolenic and linoleic acids from individual membrane lipids. The central veins of tomato leaflets were crushed using surgical forceps, leaves were cryosectioned and analyzed by two orthogonal matrix‐assisted laser desorption/ionization mass spectrometry imaging platforms for insight into lipid spatial distribution. Significant changes in lipid composition are only observed 30 min after wounding, while after 60 min lipidome homeostasis has been re‐established. Phosphatidylcholines exhibit a variable pattern of spatial behavior in individual plants. Among lysolipids, lysophosphatidylcholines strongly co‐localize with the injured zone of wounded leaflets, while, for example, lysophosphatidylglycerol (LPG) (16:1) accumulated preferentially toward the apex in the injured zone of wounded leaflets. In contrast, two other LPGs (LPG [18:3] and LPG [18:2]) are depleted in the injured zone. Our high‐resolution co‐localization imaging analyses suggest that linolenic acids are predominantly released from PCs with 16_18 fatty acid composition along the entire leaf, while it seems that in the apex zone PG (16:1_18:3) significantly contributes to the linolenic acid pool. These results also indicate distinct localization and/or substrate preferences of phospholipase isoforms in leaf tissue.

## Graphical abstract

Mass spectrometry imaging reveals consistent accumulation of specific lysolipids in wounded zones of tomato leaves.

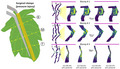

## 1 INTRODUCTION

Wounding is a ubiquitous experience of plants where its impacts on crop productivity range from herbivory to weather damage. The plant response to wounding extends from rapid vasculature occlusion (analogous to clotting; Ernst et al., 2012), to systemic protection (Delano‐Frier et al., 2013) that reaches beyond the plant through volatiles to warn other plants, and even attract allies for protection (Unsicker et al., 2009). Mechanical wounding in tomato leaves induces differential expression of more than 200 genes (Scranton et al., 2013). The first molecular signal that is released is an 18‐amino acid polypeptide hormone systemin which triggers the synthesis of powerful inducers of defense gene transcription, jasmonic acid (JA), and 12‐oxophytodienoic acid (PDA; Ali & Baek, 2020). In this lipid‐mediated signaling cascade, phospholipase enzymes play a key role in the production of JA and PDA by releasing linolenic acid from membrane phospholipids (Ali & Baek, 2020; Ryu, 2004; Vu et al., 2014). Other modifications of membrane lipids, like oxidation of fatty acids on galactolipids and head group acylation of monogalactosyldiacylglycerols (MGDG), are also reported as part of the wounding response in plant leaves (Vu et al., 2014).

Despite extensive study of the cascade of molecular events in response to plant wounding, there is very limited knowledge on the contribution and fate of individual membrane lipids (Hou et al., 2016; Vu et al., 2014; 2015), especially their spatial distribution. Bulk lipidomic mass spectrometry (MS) analyses have been employed to reveal the changes in levels of membrane lipids during the wounding process and to show that individual plants vary in lipid composition despite application of comparable stress (Vu et al., 2014; 2015). In general, it was observed that levels of structural lipids in *Arabidopsis* leaves decrease, whereas monoacyl molecular species (e.g. lysophospholipids), galactolipids, and phosphatidylglycerols increase (Vu et al., 2014). The significant limitation of these bulk analyses is that spatial information is lost, as molecular signatures are averaged across the sample, hence highly localized and low abundance lipids can be obscured in the data. The ability to observe lipid changes that occur locally at the site of injury can not only help resolve localized versus systemic lipid responses, but also provide lipid markers of leaf wounding that are mechanistically linked to plant recovery (Leon et al., 2001). Matrix‐assisted laser desorption/ionization (MALDI) mass spectrometry imaging (MSI) is a robust chemical imaging technology that uses a focused laser beam to ablate and ionize material into the mass spectrometer at high spatial resolution. Combined with spatial probing, a MALDI imaging experiment enables simultaneous visualization of hundreds of molecules mapped to tissue morphology. This approach has been widely used to localize injury‐induced lipid changes in the brain (Hankin et al., 2011; Mallah et al., 2018; 2019) and other mammalian organs (Quanico et al., 2018; Rao et al., 2016), as well as map lipid profiles in plant tissues affected by biotic or abiotic stresses (Sarabia et al., 2018).

Here, we applied global lipidomic and multimodal MALDI‐MSI analyses to characterize spatial changes in membrane lipids of tomato leaves harvested 30 min after mechanical wounding of the primary vein. We used three biological replicates: three pairs of tomato leaflets harvested from the three individual plants. Each pair is composed of a wounded and a control leaflet and each leaflet represented with a cross‐section from the apex (tip), middle, and base portion of the organ. Micrometer scale resolution of biological replicates enabled visualization of individual plant response to wounding, to reveal consistent patterns of spatial‐chemical lipid changes during the wounding process.

## 2 MATERIALS AND METHODS

### 2.1 Sample preparation

The central vein of 18 primary leaflets from six tomato plants (*Solanum lycopersicum*, purchased at the local market) were crushed using surgical forceps, and 9 leaflets were harvested 30 min (T30) and nine leaflets 60 min (T60) after the injury. Before wounding, 18 control primary leaflets were harvested on the opposite side of the rachis (Figure S1).

### 2.2 Liquid chromatography‐tandem mass spectrometry (LC‐MS/MS) analysis

For global lipidomic analysis, 15 ml Falcon tubes were pre‐weighted and an apex, middle or base section of the leaflet was dissected and snap‐frozen in liquid nitrogen. In total, there were 108 samples (18 control × 3 segments + 9 T30 × 3 segments + 9 T60 × 3 segments). Lipids were extracted using chloroform‐methanol and analyzed on a Linear Trap Quadrupole‐Orbitrap Velos (Thermo Fisher Scientific; Methods S1). Two samples were removed from our analysis (one control sample and one 30 min after wounding sample due to poor data quality), resulting in 106 analyzed samples. LIQUID software (Kyle et al., 2017) was used for confident lipid identification on the fatty acyl/alkyl level. Figures and text apply LIPID MAPS Classification, Nomenclature and Shorthand Notation for MS‐derived Lipid Structures (Liebisch et al., 2020).

### 2.3 MALDI MS imaging

**FIGURE 1:**
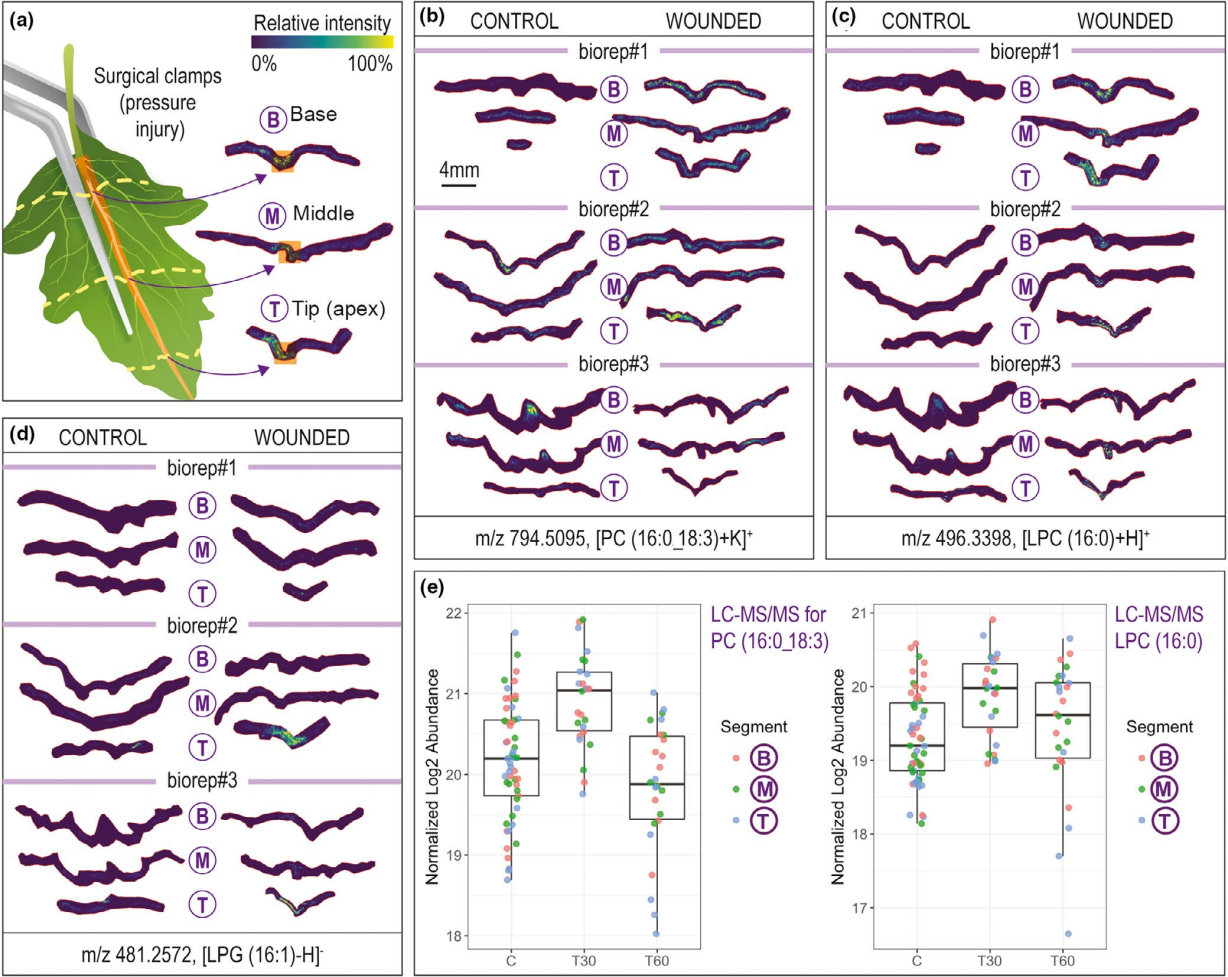
Random and preserved lipid spatial changes during tomato leaflet midrib wounding revealed by matrix‐assisted laser desorption/ionization (MALDI) 15T‐Fourier transform ion cyclotron resonance MS imaging analyses on bio‐replicates. Pixel size of all ion images is 75 µm × 75 µm. Ion images of individual m/z values were generated on the same colorbar scale for visual comparison in terms of relative ion abundance. (a) Wounding of a leaflet through the central vein and location of the base (B), middle (M), and apex (tip; T) cross‐sections used for MALDI MSI analysis. (b) MALDI ion images of m/z 794.5095, corresponding to phosphatidylcholine (PC) (16:0_18:3) + K^+^, in three biological replicates of tomato control and wounded leaves illustrate an example of a lipid with random spatial distribution in individual plants. (c) MALDI ion images of m/z 496.3398, corresponding to lysophosphatidylcholine (LPC) (16:0) + H^+^, in three biological replicates of tomato control and wounded leaves, illustrate an example of a lipid with preserved spatial distribution in individual plants with accumulation in midrib wounding zone. (d) Distribution of lysophosphatidylglycerol (LPG) (16:1) in 3 biological replicates, represented with m/z 481.2572 ion images, shows the accumulation of this lipid in the wounding zone, but only in the tip section of the tomato leaf. (e) Bulk lipidomic data indicate that on average there is a significant increase of PC (16:0_18:3) and LPC (16:0) 30 min after wounding compared to controls (PC [16:0_18:3], *p* = .0164; LPC [16:0], *p* = .0259). Boxplots of LC‐MS analyses indicate higher inter‐segment and inter‐sample variability in PC (16:0_18:3) abundance compared to LPC (16:0) abundance

For MALDI MSI, the three control and three T30 wounded leaflets were harvested whole and snap‐frozen in 50 ml Falcon tubes filled with 2.5% carboxymethyl cellulose to preserve leaflet orientation. The leaves were cryosectioned (Cryostar NX70; Thermo Fisher Scientific) perpendicular to the vein direction for the collection of the apex, middle and base sections (30 µm thickness; Figure 1a). Sections were thaw‐mounted on indium tin oxide coated glass slides (Bruker Daltonics), sprayed with MALDI matrix using TM‐sprayer M3 (HTX Technologies LLC), and analyzed on two Bruker MALDI imaging platforms: Solarix 15T‐FTICR (with 2,5‐dihydroxybenzoic acid [DHB] in positive mode and norharmane matrix in negative mode) and timsTOF fleX (with DHB matrix in positive mode). A detailed description of MALDI matrix application conditions and MALDI‐MS imaging conditions are provided in Supporting Information (Methods S1).

### 2.4 Statistical analyses

For each lipid identified by LC‐MS/MS, a mixed‐effects ANOVA was run with fixed effects of treatment and leaf segment and the interaction of treatment and leaf segment, and nested random effects of leaf within plant to account for samples coming from the same leaves and plants. Models were fit in R (version 3.6.1; R Core Team, 2020) using the lmer() function from the *lme4* package (Bates et al., 2015). A Wald test for a significant interaction term, using Type III sums of squares, was first investigated to determine if the effect of treatment differed between leaf segments. If the interaction term was not significant, the pairwise comparisons of T30 versus control and T60 versus control were run across all segments. If the interaction term was significant, the pairwise comparisons of T30 versus control and T60 versus control were run within each leaf segment. In both cases, a Tukey multiple comparison adjustment (Kutner, 2005) was included to correct for multiple testing. SCiLS Lab software (version 2020a, Bremen, Germany) was used for image processing and creating m/z co‐localization (based on Pearson's correlation analysis) plots. Imaging experiments (datasets) of wounded and control leaf segments were combined into single imaging SCiLS (.slx) file so that relative intensities of the individual ion image over control and wounded leaf segment can be compared.

## 3 RESULTS

LC‐MS/MS lipidomic experiments performed (MS/MS spectra and lipid identification can be found in Spectra S1) on extracts from control leaflets and leaflets harvested 30 and 60 min after wounding, were statistically compared across nine biological replicates for each condition. Two samples were removed from our analysis (one control sample and one 30 min after wounding sample due to poor data quality), resulting in 106 analyzed samples. A total of 71 and 126 lipids were observed in at least one of the 106 samples for the negative and positive ionization data, respectively. Significant changes in lipid composition are only observed 30 min (T30) after wounding, while after 60 min (T60) lipidome homeostasis has been re‐established, “recovering” has occurred, and only a few lipids still show elevated or decreased content compared to control leaflets (all lipids that showed statistically significant (*p* < .05) changes in abundance in response to wounding are listed in Table S1). For example, among lipids with alpha‐linolenic acid (18:3) and/or linoleic acid (18:2) composition, phosphatidylcholines (PCs), phosphatidylethanolamines (PEs), and triacylglycerols (TGs) are found to accumulate while sulfoquinovosyldiacylglycerols (SQDGs), diacylglycerols (DGs) and phosphatidic acids (PAs) decline in the first half‐hour after wounding. An hour after wound infliction, a few TGs (TG [16:3_18:2_18:3] and TG [16:3_18:3_18:3]) remained at an increased abundance and PE (18:2_18:2) and PG (16:0_16:1) at decreased abundance compared to control leaves. All other lipids had recovered to indistinguishable differences between control and wounded leaves.

Because these LC‐MS/MS experiments averaged data across the entire leaflet, there is a high probability that localized lipid changes in the wounding zone are overlooked and that other changes are not a consequence of signal propagation but are instead a result of sample‐to‐sample variation. Therefore, complementary in situ MALDI‐Fourier transform ion cyclotron resonance (FTICR)‐MSI and MALDI‐Trapped Ion Mobility Spectrometry Time‐of‐Flight (timsTOF)‐MSI experiments were performed across T30 wounded leaf sections when the greatest variations in lipid changes are expected to occur. FTICR‐MS, ultra‐high‐performance mass analyzer, allowed confident determination of the chemical formula of imaged lipid ions, whereas the advanced laser configuration of the timsTOF allowed higher lateral resolution images for greater spatial resolution within wounded zones of the tomato vasculature.

Many imaged lipids displayed striking plant‐to‐plant variations in spatial response to leaf injury. Lipids that were consistently identified in the MALDI‐MSI analyses over the three biological replicates can be found in Table S2. Observed plant‐to‐plant variation is best illustrated by phosphatidylcholine (PC) (16:0_18:3) in Figure 1b. Detected PC in one replicate displayed elevated levels across the entire leaflet for the wounded plant relative to control (biorep#1), where in a different replicate elevated PC was particularly pronounced for the cross‐section towards the apex of the wounded leaflet (biorep#2). A further variation is observed in biorep #3, where PC is accumulated around the primary vein only in the control leaflet. Although the bulk lipidomic data indicated an average increase of PC (16:0_18:3) 30 min after wounding, the boxplot in Figure 1e displays large variations across regions and biological replicates for individual measurements. Other lipid classes, including MGDG and SQDG, also show highly variable distribution upon wounding in individual plants (Figure S2).

**FIGURE 2:**
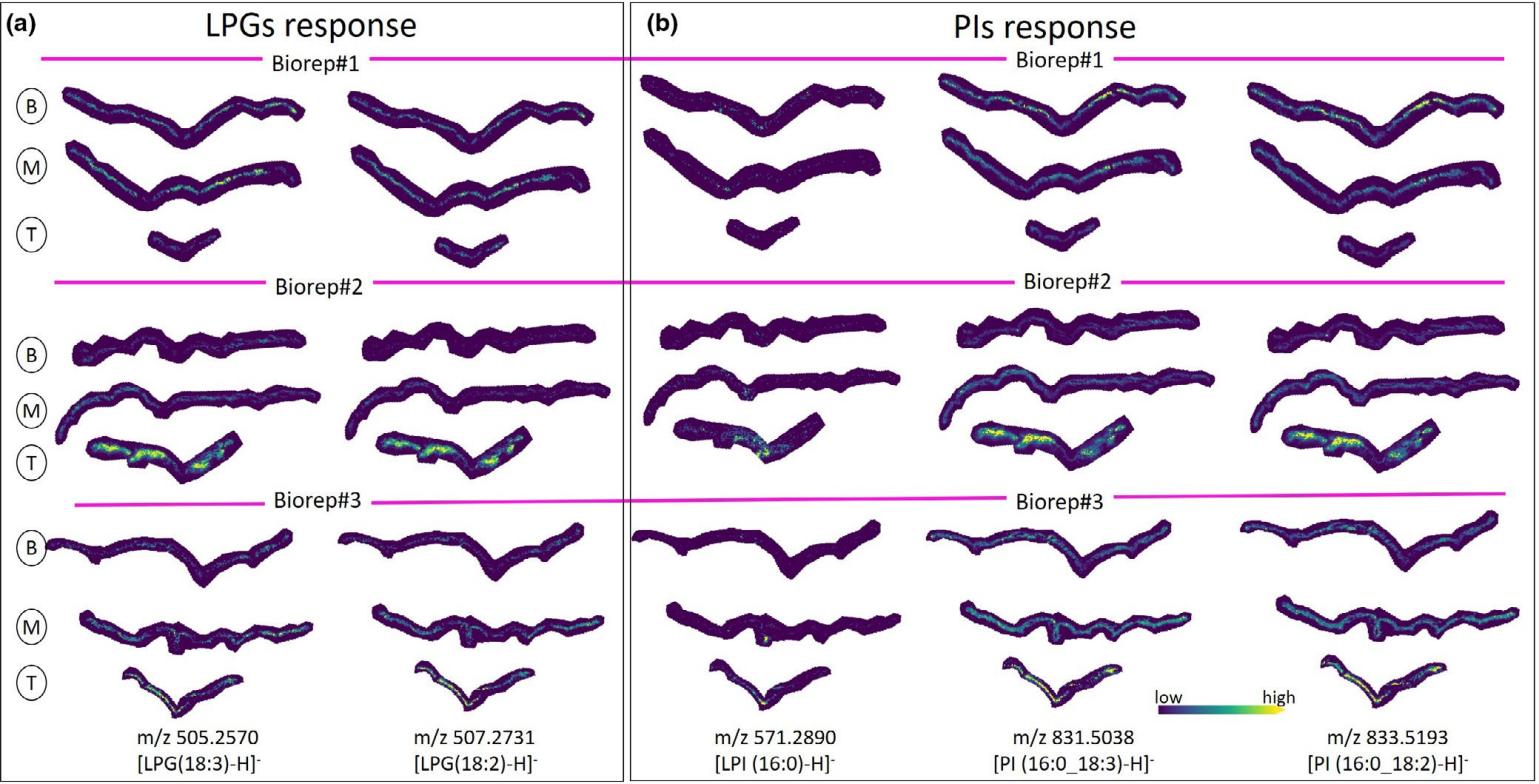
Distribution of lysolipids that are not accumulated in injured site. (a) Distribution of lysophosphatidylglycerol (LPG) (18:3) and LPG (18:2) in tomato leaflet wounded at the central midrib as revealed by matrix‐assisted laser desorption/ionization (MALDI)‐Fourier transform ion cyclotron resonance (FTICR)‐MS imaging. (b) Spatial response of PIs during tomato leaf wounding as revealed by MALDI‐FTICR‐MS imaging. Accumulation of lysophosphatidylinositol (LPI) (16:0), m/z 571.2890, in the tip and middle cross‐section of the tomato leaf (biorep#2 and biorep#3) along the midrib wounding zone. Minimal accumulation of LPI (16:0) is observed in biorep#1. PI (16:0_18:3) and PI (16:0_18:2), potential precursors of LPI (16:0), show the nearly homogeneous distribution in biorep#3 and biorep#1 (although there is depletion of the signal in the base wounded zone), while in biorep#2 there is obvious depletion of the signal in the midrib wounded zone

Several lipids displayed a consistent spatial response in all examined plants (Figure 1c ; Figure S3). All imaged lysophosphatidylcholines (LPC (16:0), LPC (18:3), and LPC (18:2)) strongly co‐localize with the injured zone of wounded leaflets and are largely absent from the control leaflets and non‐injured areas of the wounded leaves (statistically confirmed using the receiver operating characteristic (ROC): areas under the ROC curves were higher than 0.6). Similar localization was also observed for lysophosphatidylethanolamine (LPE [12:0]) and lysophosphatidic acid (LPA [18:1]; Figure S3). Not all lysolipids display accumulation around the injured zone of wounded leaves (Figure 2) possibly due to phospholipase isoforms in leaf tissue with distinct localization and/or substrate preferences (Wang, 2001). For example, the molecular response of different lysophosphatidylglycerols (LPGs) gave different behaviors, Figure 2a. LPG (16:1) accumulated preferentially toward the apex in the injured zone of wounded leaflets, (Figure 1d, bioreps 2 & 3). In contrast, two other LPGs (LPG [18:3] and LPG [18:2]) are not concentrated in the injured zone. PG (16:1_18:3) may be a preferred PLA substrate triggered by wounding to produce LPG (16:1). Linolenic acid (18:3) may be comparably produced in this event but metabolized further as part of the signaling cascade. One more lysolipid, lysophosphatidylinositol (LPI) (16:0) shows localization around the wounded area of the middle and apex region of the leaf (Figure 2b, bioreps 2 & 3). The distribution of detected PIs that include a 16:0 fatty acid component (PI [16:0_18:3] and PI [16:0_18:2]) show depletion around the wounded zone in some bio‐replicates but not others (Figure 2b).

**FIGURE 3:**
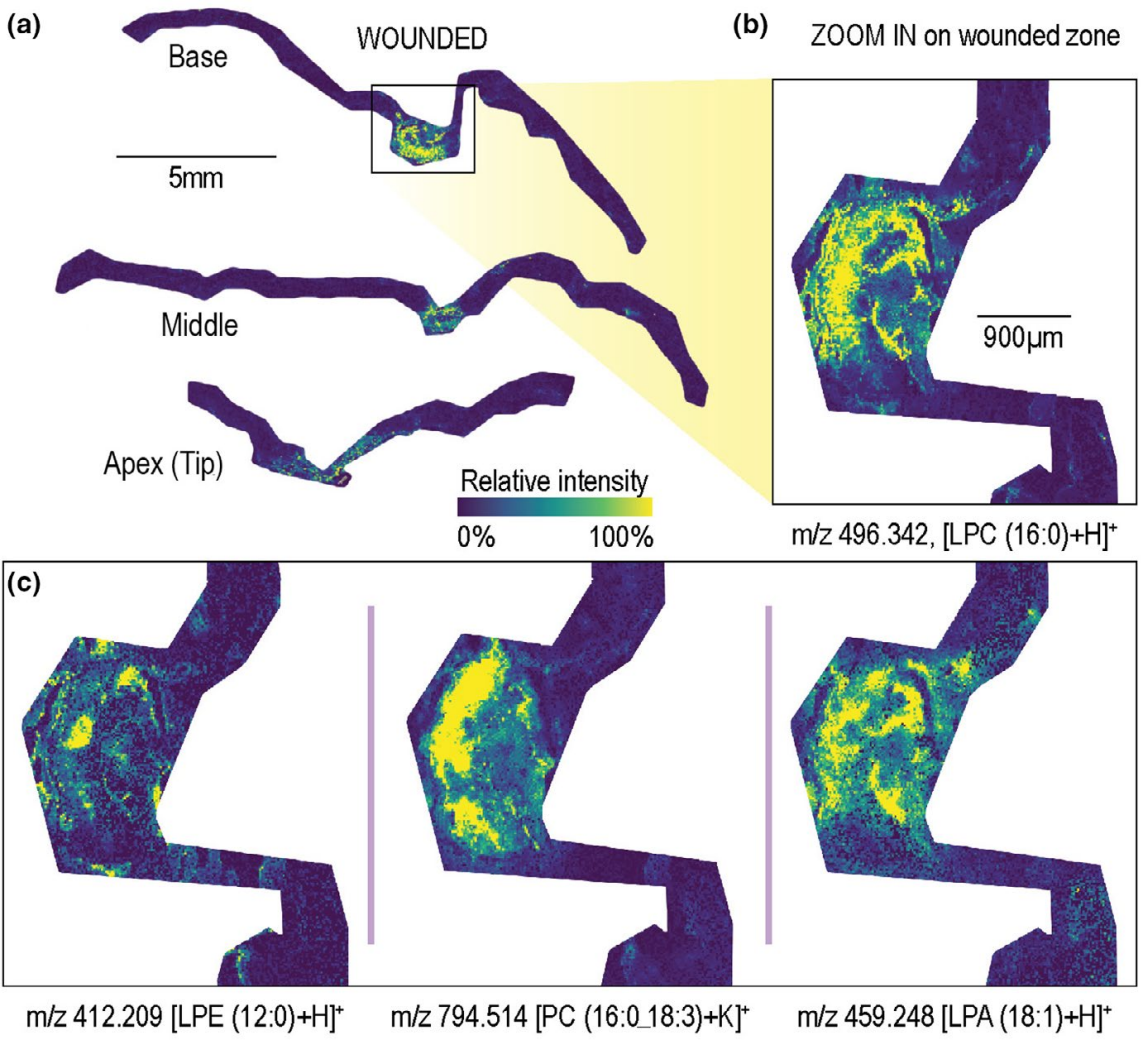
Detailed spatial distribution of phospholipids (PL) in midrib wounded leaf revealed using matrix‐assisted laser desorption/ionization‐timsTOF analysis. Pixel size is 20 µm × 20 µm. Ion images of individual m/z values were generated on the same colorbar scale for visual comparison in terms of relative ion abundance. (a) Comparison of base, middle and apex section where LPC (16:0) is observed only in the wounded zone. (b) Enlarged wounded zone of base section so that fine spatial distribution can be observed. (c) Ion images of other PLs show different spatial distribution patterns inside wounded zone

Accumulation of lysophospholipids in the wounded zones is explored in more detail by employing another MALDI imaging platform, timsTOF fleX (Figure 3; all high resolution PCs and LPCs ion images are provided in Figure S4 and S5). For example, we observe the co‐localization of all imaged LPCs (Pearson correlation coefficient [Pcc] between 0.57 and 0.79), Table 1. The highest spatial correlation (Pcc > 0.75) is between LPCs (16:0):(18:3) and LPCs (16:0):(18:2) and LPCs (16:0):(18:0) ion image pairs, while lower correlation (Pcc = 0.57–0.67) is observed between individual 18C LPCs. Notably, the bulk lipidomic analysis also revealed that LPC (16:0) and LPC (18:2) accumulated during wounding with *p*‐values .026 and .0496, respectively (Figure 1e, LPC [16:0] boxplot).

**TABLE 1:** Pearson correlation coefficients (Pcc) between lipid ion images in MALDI timsTOF MSI analysis. In general, Pcc > 0.75 reflects high co‐localization

	LPE(12:0)	LPA(18:1)	LPC(18:3)	LPC(18:2)	LPC(18:0)	LPC(16:0)	PC(18:2_18:2)	PC(18:2_18:3)	PC(18:0_18:3)	PC(18:0_18:2)	PC(16:0_18:2)	PC(16:0_18:3)
LPE(12:0)	1.000	0.033	0.079	0.145	0.195	0.218	0.005	0.004	0.006	0.016	0.017	0.012
LPA(18:1)	0.033	1.000	0.766	0.386	0.392	0.502	0.070	0.083	0.084	0.102	0.110	0.114
LPC(18:3)	0.079	0.766	1.000	0.690	0.574	0.767	0.088	0.106	0.092	0.103	0.121	0.132
LPC(18:2)	0.145	0.386	0.690	1.000	0.567	0.754	0.168	0.187	0.151	0.146	0.183	0.199
LPC(18:0)	0.195	0.392	0.574	0.567	1.000	0.788	0.097	0.096	0.095	0.139	0.147	0.136
LPC(16:0)	0.218	0.502	0.767	0.754	0.788	1.000	0.103	0.109	0.100	0.122	0.148	0.147
PC(18:2_18:2)	0.005	0.070	0.088	0.168	0.097	0.103	1.000	0.920	0.841	0.795	0.872	0.873
PC(18:2_18:3)	0.004	0.083	0.106	0.187	0.096	0.109	0.920	1.000	0.811	0.740	0.819	0.849
PC(18:0_18:3)	0.006	0.084	0.092	0.151	0.095	0.100	0.841	0.811	1.000	0.798	0.854	0.864
PC(18:0_18:2)	0.016	0.102	0.103	0.146	0.139	0.122	0.795	0.740	0.798	1.000	0.909	0.864
PC(16:0_18:2)	0.017	0.110	0.121	0.183	0.147	0.148	0.872	0.819	0.854	0.909	1.000	0.954
PC(16:0_18:3)	0.012	0.114	0.132	0.199	0.136	0.147	0.873	0.849	0.864	0.864	0.954	1.000

## 4 DISCUSSION

Observed plant‐to‐plant variations in molecular images of PCs, MGDGs and SQDGs are consistent with previous observations that individual plants under comparable treatment show biological variations in the rate and extent of activation of various metabolic pathways (Steuer et al., 2003; Vu et al., 2014). Notably, the ability to achieve experimental consistency, in precisely controlled incubators, is not indicative of the biological variation in a more realistic plant growth environment (with variations in watering, shading, etc.). Accumulation of lysophospholipids at the site of injury indicates that the production of the lysophospholipids is a direct result of cellular damage. Accumulation of these monoacyl lipids in the injured zone results from phospholipid hydrolysis by phospholipases A (PLAs) and is responsible for systemic wound signal transduction in the tomato leaf (Conconi et al., 1996; Narvaez‐Vasquez et al., 1999) and plants in general (Vu et al., 2014; 2015). Our co‐localization analysis in wounded zones suggests that substrates of signal transduction (linoleic and linolenic acids) are mostly released from PCs with 16_18 fatty acid composition. High co‐localization (Pcc = 0.81–0.96) between individual PCs in the wounded zone of each leaflet implies that there is not selective spatial partitioning of these molecules during the wounding response. Although LPE is similarly up‐regulated like LPCs in wounding zones, LPE lacks co‐localization distribution patterns (Pcc < 0.3) implying that cells involved in the wounding response have significantly altered membrane composition and/or selective PLA activity. While the specificity of PLA activity induced by wounding in plants is still not well understood (Mariani & Fidelio, 2019), our findings detailing in situ co‐localization of phospholipase products provide an analytical method to advance hypotheses to understand the role of lipid enzyme specificity on plant stress response. It is important to recognize that the MALDI‐MSI technique is measuring the relative abundance of lipids, which are the result of the dynamic production and further transformation of these dynamic cellular components. Due to phospholipid‐based signal transduction, a small number of events can elicit a robust biological response—our MSI measurements capture these subtle lipidomic changes on a µm spatial scale.

The complexity of plant lipidomic characterization presents challenges that require replication and independent, orthogonal methodologies, so that accurate assessments of lipid dynamics can be applied to our understanding of plants under stress (Vu et al., 2014). Our results highlight the importance of including high‐resolution image analysis in the interpretation of localized responses that occur during localized stress. Our multimodal MALDI‐MSI experiments characterized the lipidomic response at the site of wounding and surrounding regions by creating snapshots detailing how individual lipid species were distributed 30 min after injury. Replicate imaging revealed co‐localization of some lipid classes, and dynamic response of other lipids that while consistent with bulk lipid measurements, reveal tremendous spatial variability in response to wounding. More specifically, the co‐localization analysis provided biomolecular evidence for the pathway origin of linolenic acid—an important substrate for signal transducer biosynthesis during wounding. This unique ultra‐high‐resolution multi‐component biomolecular characterization can assist in a better understanding of PLAs activation, specificity, and selectivity in the initial steps of stress signaling biochemistry. Understanding of these signal transduction cascades at a spatial‐molecular level can aid in developing future breeding and genetic engineering strategies for plant protection from stresses due to herbivore‐attacks, pathogen infections, and environmental stresses such as drought.

## CONFLICT OF INTEREST

The authors have no conflict of interest to declare.

## Supporting information

Supplementary Material
