# Supplementary material for: Preserved and variable spatial‐chemical changes of lipids across tomato leaves in response to central vein wounding reveals potential origin of linolenic acid in signal transduction cascade
Source: Plant Environ Interact. 2021 Feb 1;2(1):28–35. doi: 10.1002/pei3.10038 (PMC10168036; doi:10.1002/pei3.10038)
Supplement: Supplementary file 1 — Supplementary Material [file PEI3-2-28-s001.docx]

**Supporting information**

**Article title:** Preserved and variable spatial-chemical changes of lipids across tomato leaves in response to central vein wounding reveals potential origin of linolenic acid in signal transduction cascade

**Authors:** Dusan Velickovic, Rosalie Chu, Corinna Henkel, Annika Koch, Nannan Tao, Jennifer E. Kyle, Joshua N. Adkins, Christopher Anderton, Vanessa Paurus, Kent Bloodsworth, Lisa M. Bramer, Shannon Cornett, Wayne Curtis, Kristin E. Burnum-Johnson

The following Supporting Information is available for this article:

**Fig. S1** Selection of control (zero minutes (T0)) and wounded (thirty minutes (T30) or sixty minutes (T60)) leaflets segments from tomato plant for LC-MS/MS and MALDI analysis.


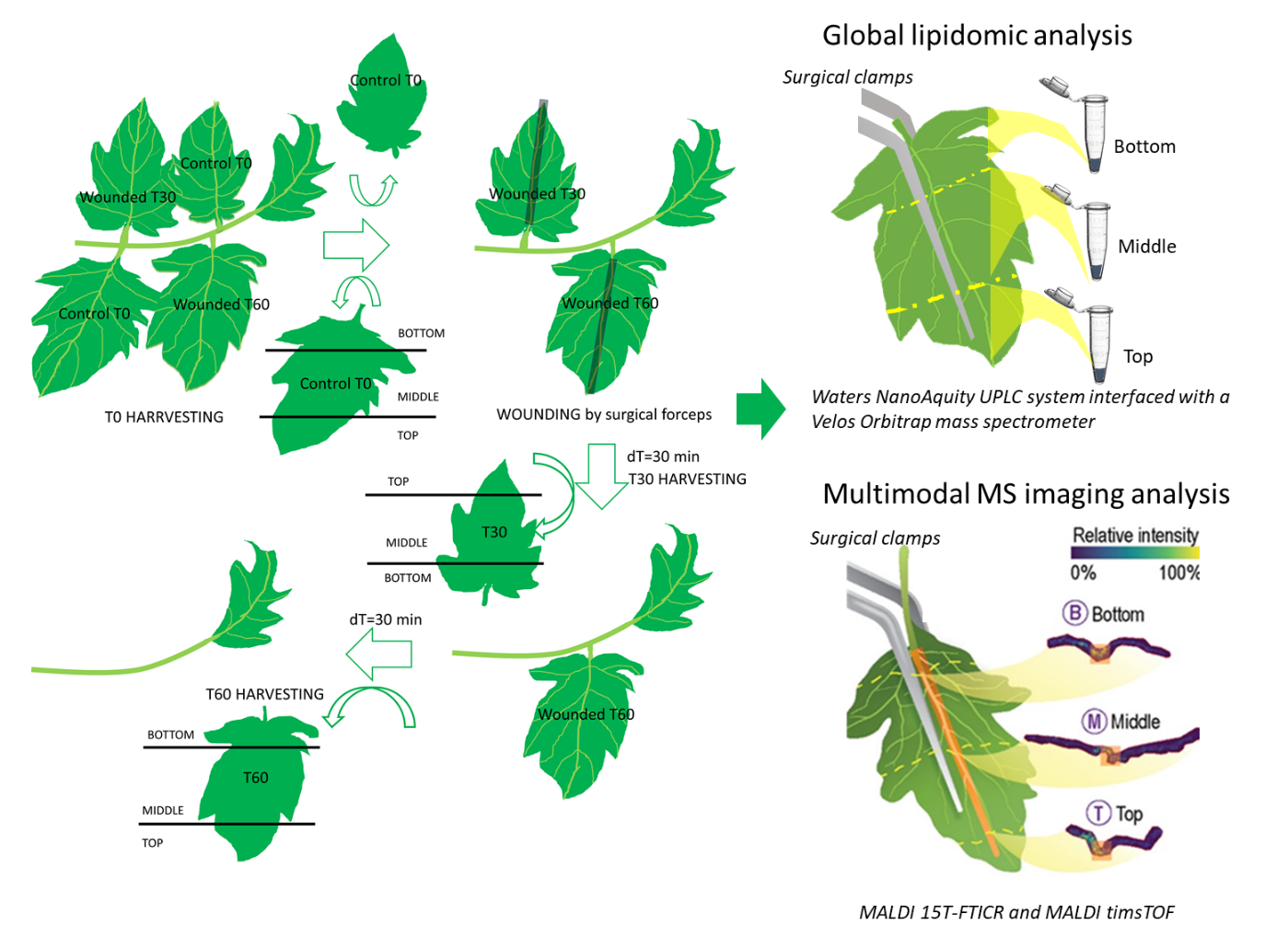


**Fig. S2** MALDI-FTICR-MS ion images that show the spatial distribution of MGDG and SQDG revealed through 3 biological replicates in base (B), middle (M) and tip (T) section of the wounded and control tomato leaf. Note that at least one bio-replicate shows different patterns. For example, biorep#1 shows that MGDG is upregulated in all areas of tomato leaves except in the wounded zone, which is not cased with the other 2 bio-replicates. Bio-rep #2 shows increased expression of SQDG in the tip part of the wounded leaf out of the wounded zone.


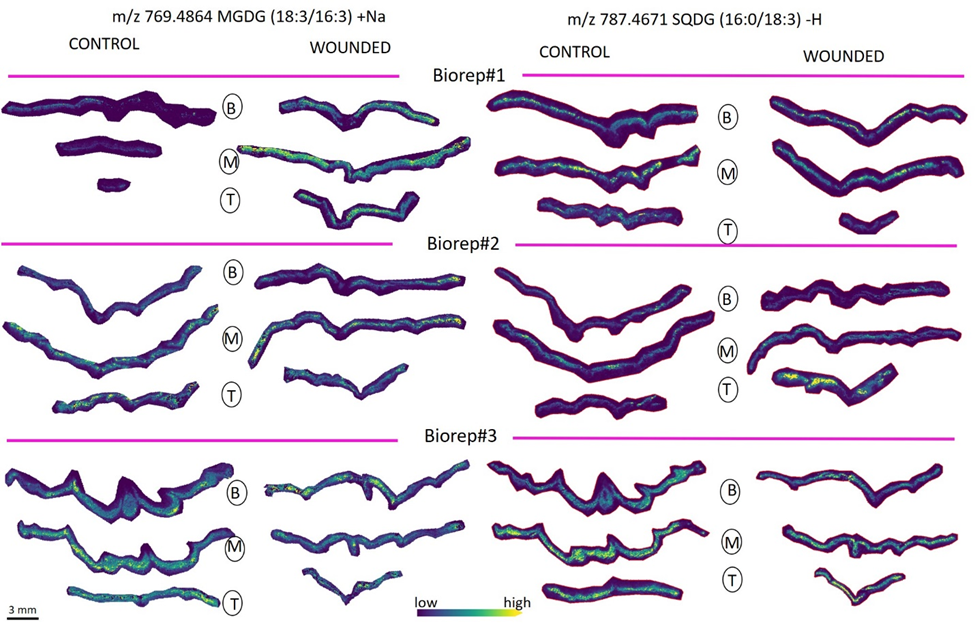


**Fig. S3** MALDI-FTICR-MS ion images show the spatial distribution of lyso-phospholipids that are accumulated in the wounded zone of tomato leaf.


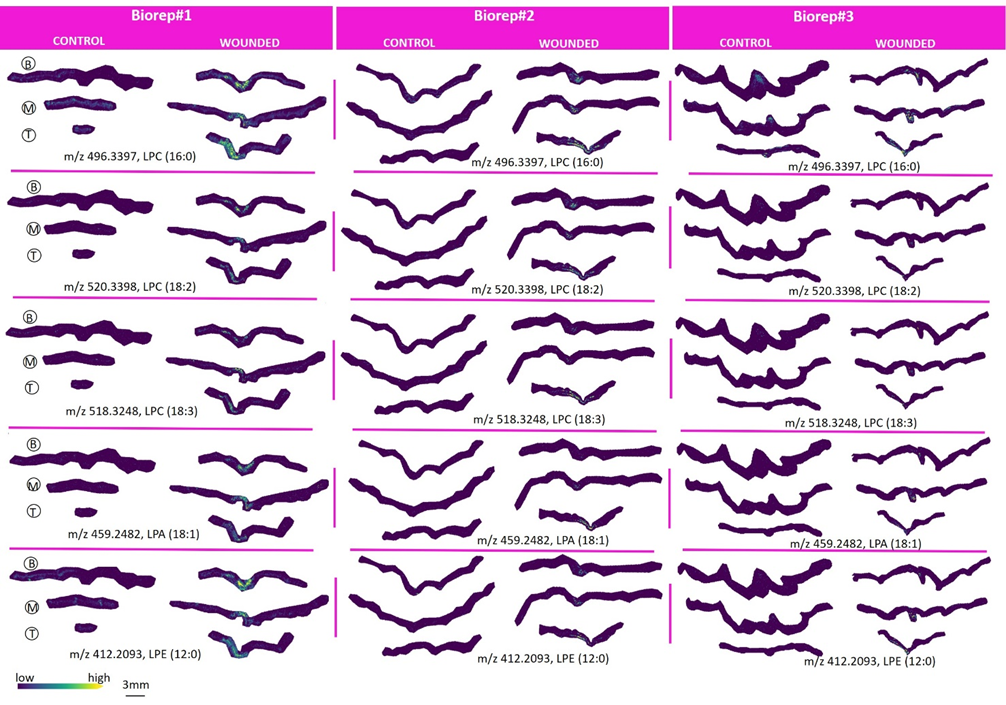


**Fig. S4** MALDI-timsTOF ion images that show the spatial distribution of lyso-phosphatidylcholines (LPC) in the wounded zone of tomato leaf. One imaging pixel is 20 µm x 20 µm.


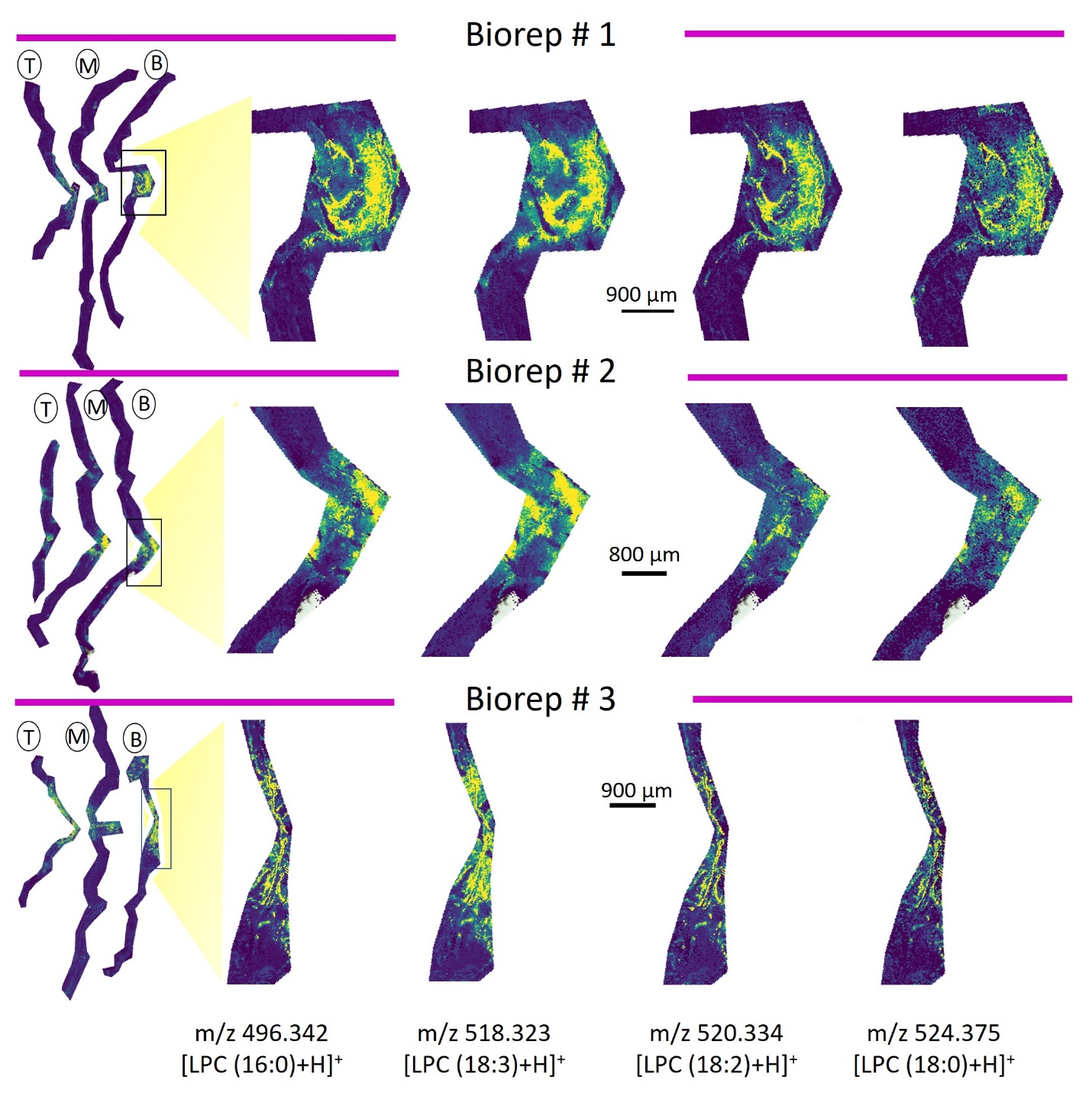


**Fig. S5** MALDI-timsTOF ion images that show the spatial distribution of phosphatidylcholines (PC) in the wounded zone of tomato leaf. One imaging pixel is 20 µm x 20 µm.


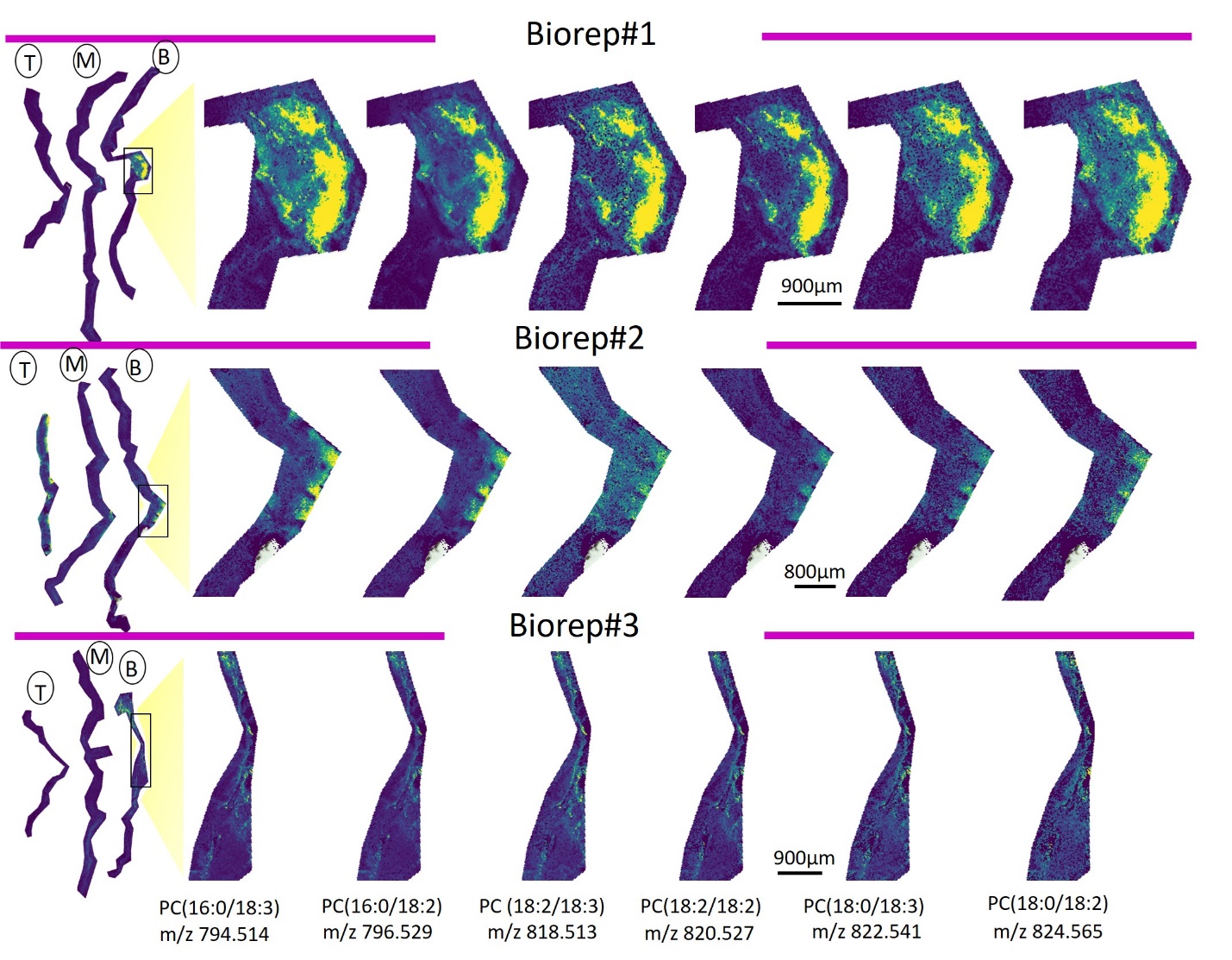


Fig. S6. MALDI ion images of two serial sections recorded on two different MALDI imaging platforms, illustrating the reliability of molecular spatial information. In both sections data show that LPC is accumulated in the wounded zone, MGDG has hot spot localization at the left “hand” of the section, tomatine, glycoalkaloid characteristic for plant leaf, is almost uniformly distributed throughout the tissue and DHB shows the highest intensities outside of the tissue.


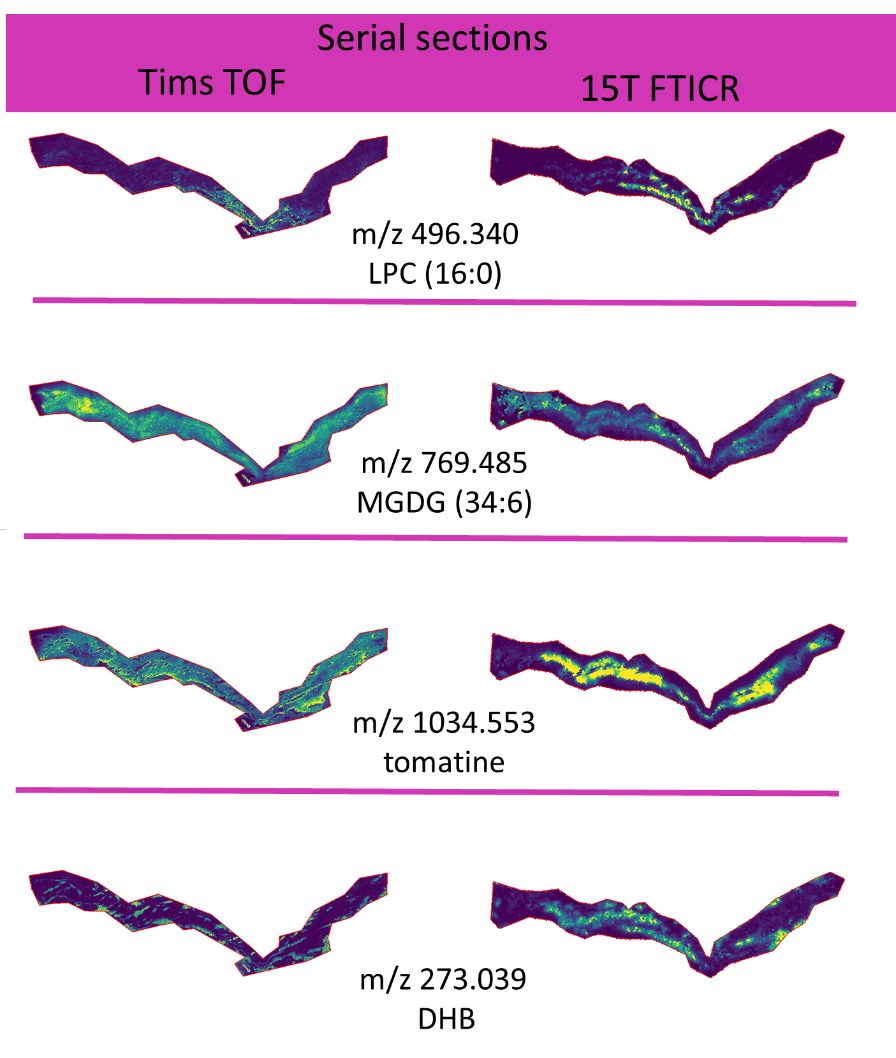


**Table S1** Lipids that showed a significant difference (p < 0.05) in abundance between wounded (30 min and 60 min after wounding) and control leaf (T0) in LC-MS/MS lipidomic study.

| **Lipid ID** | **T30 vs T0** | | **T60 vs T0** | |
| --- | --- | --- | --- | --- |
|  | **regulated** | **p value** | **T60** | **p value** |
| DG(16:0_18:1) | down | 0.0327 | N/A | >0.05 |
| DG(16:0_18:2) | down | 0.0058 | N/A | >0.05 |
| DG(17:0_18:3) | down | 0.0385 | N/A | >0.05 |
| DG(18:2_18:3) | down | 0.0363 | N/A | >0.05 |
| DG(20:0_22:0) | down | 0.0213 | N/A | >0.05 |
| LPA(16:0) | down | 0.0325 | N/A | >0.05 |
| PA(16:0_16:0) | down | 0.0008 | N/A | >0.05 |
| PA(16:0_16:1) | down | 0.0038 | N/A | >0.05 |
| PA(16:0_18:1) | down | 0.0133 | N/A | >0.05 |
| PA(16:0_18:2) | down | 0.0108 | N/A | >0.05 |
| PA(16:0_18:3) | down | 0.0356 | N/A | >0.05 |
| PA(16:1_18:3) | down | 0.0109 | N/A | >0.05 |
| PA(16:3_16:0) | down | 0.0335 | N/A | >0.05 |
| PA(17:0_18:2) | down | 0.0076 | N/A | >0.05 |
| PA(17:0_18:3) | down | 0.0002 | N/A | >0.05 |
| LPA(18:2) | down | 0.0188 | N/A | >0.05 |
| PA(18:2_18:2) | down | 0.0151 | N/A | >0.05 |
| PA(18:2_18:3) | down | 0.0263 | N/A | >0.05 |
| PA(18:2_20:2) | down | 0.0014 | N/A | >0.05 |
| PA(18:3_20:0) | down | 0 | N/A | >0.05 |
| LPC(16:0) | up | 0.0259 | N/A | >0.05 |
| PC(16:0_18:3) | up | 0.0164 | N/A | >0.05 |
| LPC(18:2) | up | 0.0496 | N/A | >0.05 |
| PC(18:2_18:2) | up | 0.0193 | N/A | >0.05 |
| PC(18:2_18:3) | up | 0.0218 | N/A | >0.05 |
| PC(18:3_18:3) | up | 0.0044 | N/A | >0.05 |
| LPE(16:0) | up | 0.0008 | N/A | >0.05 |
| PE(16:0_18:2) | up | 0.0334 | N/A | >0.05 |
| PE(16:0_18:3) | up | 0.0121 | N/A | >0.05 |
| PE(18:0_18:2) | up | 0.0212 | N/A | >0.05 |
| LPE(18:2) | up | 0.0038 | N/A | >0.05 |
| PE(18:2_18:2) | up | 0.0125 | down | 0.018 |
| PE(18:2_18:3) | up | 0.0014 | N/A | >0.05 |
| LPI(16:0) | down | 0.0334 | N/A | >0.05 |
| SQDG(16:0_16:0) | down | 0.0023 | N/A | >0.05 |
| SQDG(17:0_18:3) | down | 0.0069 | N/A | >0.05 |
| SQDG(18:0_18:3) | down | 0.0226 | N/A | >0.05 |
| TG(50:3) | down | 0.001 | N/A | >0.05 |
| TG(50:2) | down | 0.0005 | N/A | >0.05 |
| TG(16.3_18.2_18.3) | up | 0.0009 | up | 0.0018 |
| TG(16.3_18.3_18.3) | up | 0.0005 | up | 0.0026 |
| TG(18:3_18:2_18:3) | up | 0.0191 | N/A | >0.05 |
| TG(50.5) | down | 0.0321 | N/A | >0.05 |
| TG(52.3) | down | 0.0128 | N/A | >0.05 |
| PG(16:0_16:1) | N/A | >0.05 | down | 0.0454 |

| **Table S2.** Lipid ions measured consistently in all three biological replicates of tomato leaf during MALDI-FTICR-MS imaging analysis. Regulation and localization of regulation (B-base, M-middle, T-tip) of these lipids during wounding treatment are provided. | | | | |
| --- | --- | --- | --- | --- |
| Lipid | measuredm/z | Formula | MALDI MSI | |
|  |  |  | regulated | localization |
| MGDG (34:6) * | 769.4864 | C43H70O10 +Na | Random |  |
| MGDG (36:6) ^#^ | 797.5169 | C45H74O10 +Na | Random |  |
| MGDG (18:2_18:3) | 799.5338 | C45H76O10 +Na | Random |  |
| LysoPE (12:0) | 412.2093 | C17H34NO8P +H | UP | B, M, T Wound area |
| LysoPA (18:1) | 459.2482 | C21H41O7P +Na | UP | B, M, T Wound area |
| PA (16:0_18:3) | 693.4462 | C37H67O8P +Na | Random |  |
| PA (36:4) | 719.4619 | C39H69O8P +Na | Random |  |
| PA (18:0_18:3) | 721.4778 | C39H71O8P +Na | NO |  |
| LysoPC (16:0) | 496.3397 | C24H50NO7P +H | UP | B, M, T Wound area |
| LysoPC (18:3) | 518.3248 | C26H48NO7P +H | UP | B, M, T Wound area |
| LysoPC (18:2) | 520.3398 | C26H50NO7P +H | UP | B, M, T Wound area |
| PC (16:0_18:3) | 794.5097 | C42H78NO8P +K | Random |  |
| PC (16:0_18:2) | 796.5253 | C42H80NO8P +K | Random |  |
| PC (18:3_18:3) | 816.4940 | C44H76NO8P +K | Random |  |
| PC (18:2_18:3) | 818.5097 | C44H78NO8P +K | Random |  |
| PC (18:2_18:2) | 820.5253 | C44H80NO8P +K | Random |  |
| PC (18:0_18:3) | 822.5410 | C44H82NO8P +K | Random |  |
| PC (18:0_18:2) | 824.5566 | C44H84NO8P +K | Random |  |
| PG (16:1_18:3) | 765.4678 | C40H71O10P +Na | NO |  |
| PG (16:0_18:3) | 767.4826 | C40H73O10P +Na | NO |  |
| PS (P-28:0) | 664.4548 | C34H66NO9P +H | NO |  |
| PS (44:10) | 884.5442 | C50H78NO10P +H | Random |  |
| PI (42:6) | 977.5509 | C51H87O13P +K | NO |  |
| LysoPA (16:0) | 409.2363 | C19H39O7P -H | UP | T |
| LysoPA (18:2) | 433.2363 | C21H39O7P -H | UP | T |
| LysoPA (18:3) | 431.2206 | C21H37O7P -H | UP | T |
| PA (16:1_18:3) | 667.4347 | C37H65O8P -H | NO |  |
| PA (16:0_18:3) | 669.4504 | C37H67O8P -H | NO |  |
| PA (16:0_18:2) | 671.4659 | C37H69O8P -H | NO |  |
| PA (18:3_18:3) | 691.4349 | C39H65O8P -H | NO |  |
| PA (18:2_18:3) | 693.4501 | C39H67O8P -H | NO |  |
| PA (18:2_18:2) | 695.4661 | C39H69O8P -H | NO |  |
| LysoPG (16:1) | 481.2572 | C22H43O9P -H | UP | T wound area |
| LysoPG (18:3) | 505.257 | C24H43O9P -H | Random |  |
| LysoPG (18:2) | 507.2731 | C24H45O9P -H | Random |  |
| PG (16:0_16:1) | 719.4872 | C38H73O10P -H | NO |  |
| PG (16:0_16:0) | 721.5032 | C38H75O10P -H | NO |  |
| PG (16:1_18:3) | 741.4721 | C40H71O10P -H | NO |  |
| PG (16:0_18:3) | 743.4877 | C40H73O10P -H | NO |  |
| PG (16:0_18:2) | 745.5033 | C40H75O10P -H | Random |  |
| LysoPI (16:0) | 571.2890 | C25H49O12P -H | UP | T, M wound area |
| LysoPI (22:2) | 651.3515 | C31H57O12P -H | UP | T |
| LysoPI (22:1) | 653.3673 | C31H59O12P -H | NO |  |
| PI (O-32:1) | 793.5239 | C41H79O12P -H | NO |  |
| PI (O-34:4) | 815.5086 | C43H77O12P -H | Random |  |
| PI (O-34:3) | 817.5245 | C43H79O12P -H | Random |  |
| PI (16:0_18:3) | 831.5038 | C43H77O13P -H | Random |  |
| PI (16:0_18:2) | 833.5193 | C43H79O13P -H | Random |  |
| SQDG (16:0_16:3) | 787.4671 | C41H72O12S -H | Random |  |
| SQDG (16:0_16:2) | 789.4828 | C41H74O12S -H | Random |  |
| SQDG (16:0_16:1) | 791.4994 | C41H76O12S -H | Random |  |
| SQDG (16:0_16:0) | 793.5146 | C41H78O12S -H | NO |  |
| SQDG (16:1_18:3) | 813.4828 | C43H74O12S -H | Random |  |
| SQDG (16:0_18:3) | 815.4984 | C43H76O12S -H | Random |  |
| SQDG (17:0_18:3) | 829.5141 | C44H78O12S -H | Random |  |
| SQDG (36:6) | 837.4828 | C45H74O12S -H | Random |  |
| SQDG (38:6) | 839.4986 | C45H76O12S -H | Random |  |
| SQDG (18:0_18:3) | 843.5297 | C45H80O12S -H | Random |  |

** based on LC-MS structure of MGDG (34:6) can be MGDG (18:3_16:3) and MGDG (16:2_18:4)*

*# based on LC-MS structure of MGDG (36:6) can be MGDG (18:3_18:3) and MGDG (18:2_18:4).*

**Methods S1** Detailed description of the sample preparation, MALDI matrix application conditions, LC-MS, and MALDI MSI analyses parameters.

**Sample preparation for MS analyses.** The central vein of 18 primary leaflets from 6 tomato plants (*Solanum lycopersicum*, purchased at the local market) were crushed using surgical forceps, and leaves were harvested 30 min and 60 min after the injury. Before wounding, control primary leaflets were harvested on the opposite side of the rachis (Supporting information Fig. S1). For global lipidomic analysis, 15 ml Falcon tubes were pre-weighted and an apex, middle or base section of the leaflet was dissected and snap-frozen in liquid nitrogen. Lipids were extracted using chloroform-methanol and analyzed on a Linear Trap Quadrupole-Orbitrap Velos (Thermo Fisher Scientific, Bremen, Germany) (Supporting information Methods S1). For MALDI MSI the control and wounded leaves were harvested whole and snap-frozen in 50 ml Falcon tubes filled with 2.5% carboxymethyl cellulose (CMC) to preserve leaflet orientation. The leaves were cryosectioned (Cryostar NX70, Thermo Fisher Scientific) perpendicular to the vein direction for the collection of the apex, middle and base sections (30 µm thickness). Sections were thaw-mounted on indium tin oxide (ITO) coated glass slides (Bruker Daltonics, Bremen, Germany), sprayed with MALDI matrix using TM-sprayer M3 (HTX Technologies LLC, Carrboro, USA) and analyzed on two Bruker MALDI imaging platforms: Solarix 15T-FTICR (with 2,5-dihydroxybenzoic acid (DHB) in positive mode and norharmane matrix in negative mode) and timsTOF fleX (with DHB matrix in positive mode). SCiLS Lab software (version 2020a, Bremen, Germany) was used for image analysis

**MALDI matrix application.** MALDI matrix application was performed using a TM3-Sprayer (HTX Technologies). DHB (2, 5-dihydroxybenzoic acid) and norharmane were used for positive and negative ion modes, respectively. For DHB, 40 mg/mL in 50% MeOH was sprayed with 16 passes at 50 µL/min at 80 °C with spray spacing of 3 mm. For norharmane, 7 mg/mL in CHCl_3_: MeOH (2:1) was used, and seven passes were sprayed at 120 µL/min and 30 °C, with a spray spacing of 2 mm. A spray pressure of 10 psi (N2), a spray velocity of 1200 mm/min, and a sprayer nozzle distance from the sample of 40 mm was maintained for all samples.

**MALDI FTICR MSI.** MSI was performed on a 15 tesla MALDI-FTICR-MS (Bruker Daltonics) equipped with SmartBeam II laser source (355 nm, 2 kHz) in positive mode using 200 shots/pixel with a frequency of 2 kHz and a 75 µm pitch between pixels. FTICR-MS was operated to collect 250-2,000 m/z, using a 577 ms transient, which translated to a mass resolution of R 170,000 ~ at 400 m/z. Data was acquired using FlexImaging (v 4.1, Bruker Daltonics), and image processing and visualization were performed using SCiLS Lab2020a (Bruker Daltonics).

**MALDI timsTOF MSI.**

MSI was performed on a timsTOF flex (Bruker Daltonics) equipped with a SmartBeam 3D laser (355 nm, 10 kHz) in positive ion mode allowing for true pixel imaging. Mass spectra were collected in the range 100-1500 m/z using 400 shots/pixel with a frequency of 10 kHz and a 20 µm pitch between pixels using the beam scan option. Data was acquired using FlexImaging (v 5.1, Bruker Daltonics), and image processing and visualization were performed using SCiLS Lab 2020a (Bruker Daltonics).

### **Total lipid extraction.** The collected material was lyophilized for untargeted lipidomics analysis. Lipids were extracted using a modified Folch extraction (Folch et al., 1957). To break up the biological material, an average of 9 mg of sample was bead beaten using a 3 mm tungsten carbide bead in 750 µL of methanol for 2 min at a frequency of 30 Hz. The sample was then removed and transferred into a 20 mL clean EPS glass vial with a Teflon lined cap. Another 750 µL of methanol was added for a final volume of 1.5 mL methanol. Next, 3 mL of chloroform and 200 µl of water were added to each sample. The samples were vortexed for 30 s, sonicated for 30 min, vortexed again for 30 s, and then 0.925 µL of water was added to induce phase separation. The samples were incubated at 4 °C overnight and then the lower lipid layer was removed, dried down, and stored at -20 °C at a concentration of 2 µg/µL in 2:1 chloroform/methanol until Mass Spectrometry (MS) analysis.

### **Liquid chromatography (LC)-tandem mass spectrometry (MS/MS) analyses.** All extracted lipids in this manuscript were analyzed by LC-MS/MS using a Waters NanoAquity UPLC system interfaced with a Velos Orbitrap mass spectrometer (Thermo Scientific, San Jose, CA) as outline in (Kyle et al., 2017). The total lipid extracts (TLE) were reconstituted in methanol for a final abundance of 0.4 µg TLE/µL. Ten µL of the TLE were injected onto Waters column (HSS T3 1.0 mm x 150 mm x 1.7 µm particle size). Lipids were separated over a 34 min gradient elution (mobile phase A: ACN/H_2_O (40:60) containing 10 mM ammonium acetate; mobile phase B: ACN/IPA (10:90) containing 10 mM ammonium acetate) at a flow rate of 250 µL/min. Samples were analyzed in both positive and negative ionization using HCD (higher-energy collision dissociation) and CID (collision-induced dissociation) to obtain high coverage of the lipidome.

**Lipid identification and alignment.** We used LIQUID software(Kyle et al., 2017) for confident lipid identification. All LC-MS/MS data were aligned and gap-filled based on the identified lipid name, observed m/z, and the retention time (grouped by sample type and ionization mode) and matching unidentified features to their identified counterparts using MZmine2(Pluskal et al., 2010). Aligned features were manually verified and peak apex intensity values are exported for statistical analysis.

**Spectra S1.** MS/MS spectra of lipids described in the manuscript and their identification using LIQUID software. CID and/or HCD spectrum are present depending on fragments intensities.

**MGDG (34:6)-identified as MGDG (18:3_16:3) or MGDG (16:2_18:4)**

**
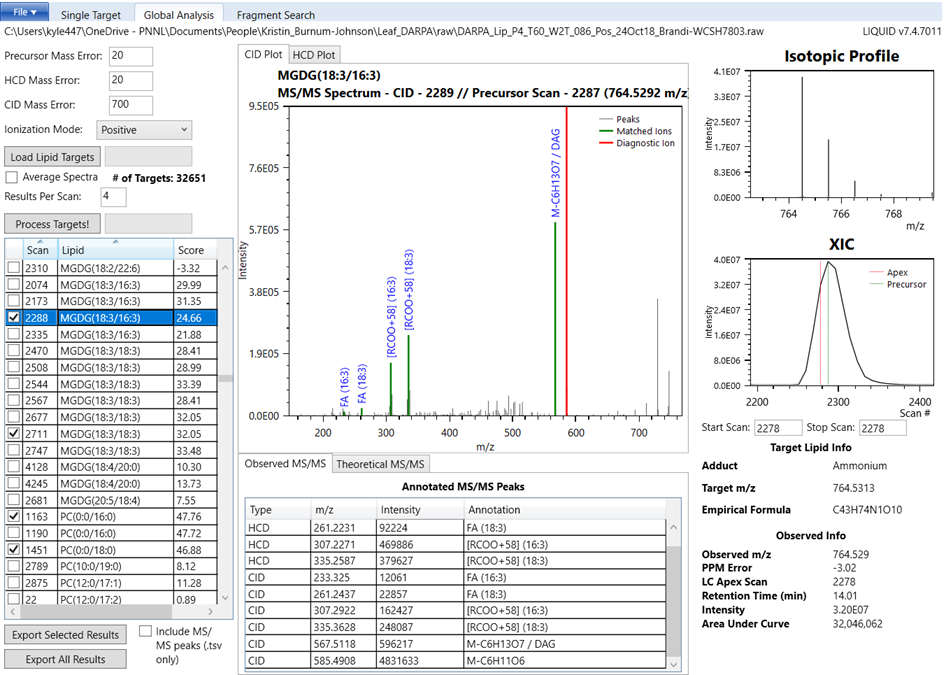
**

**
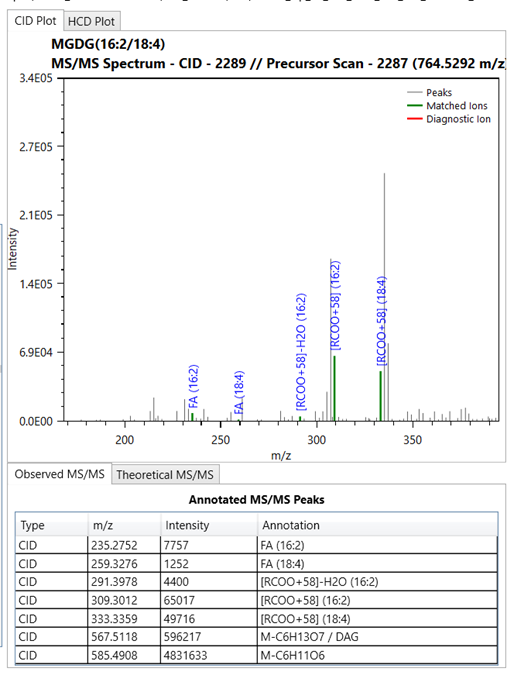
**

**LPC (16:0)**

**
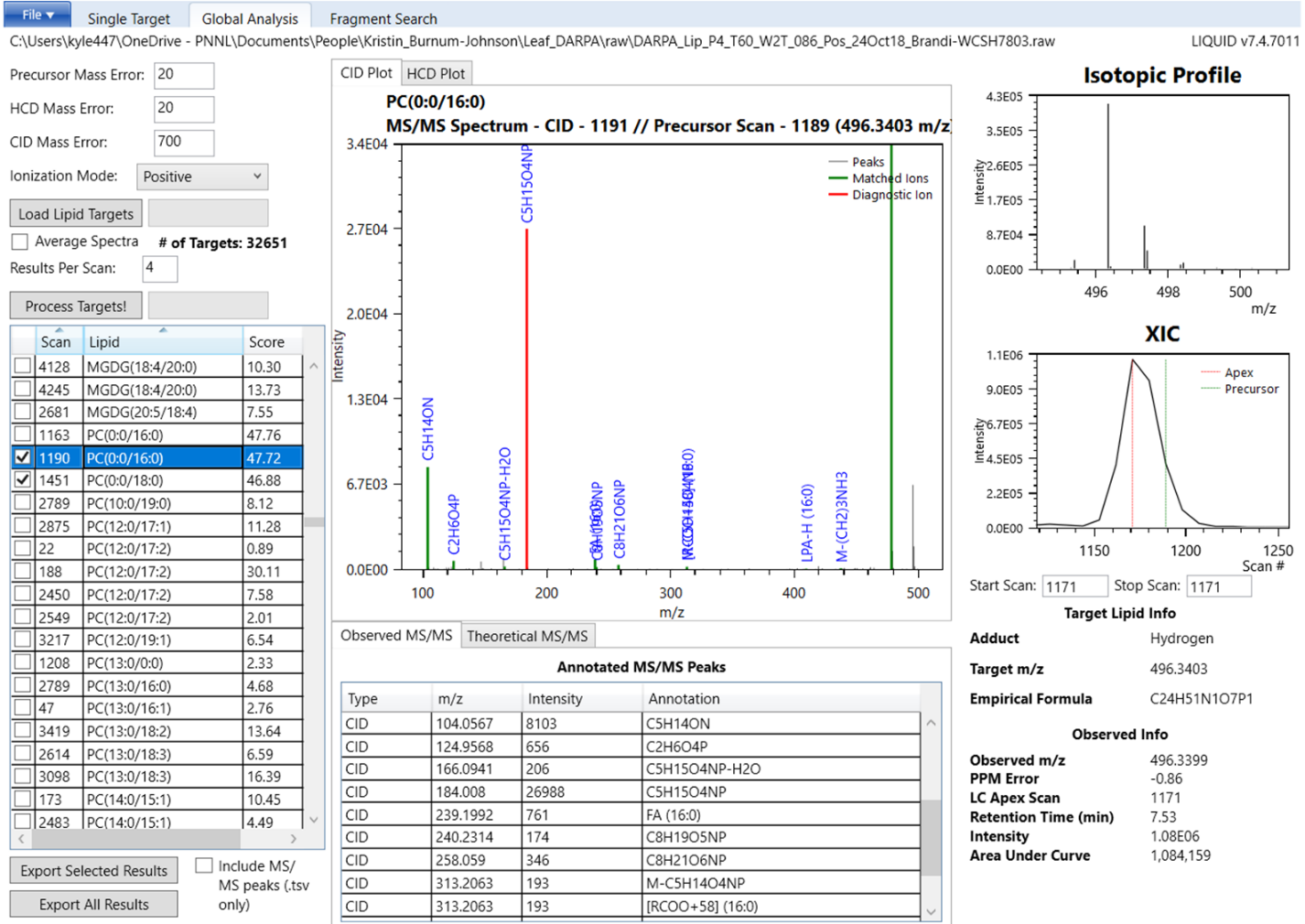
**

**LPC (18:2)**

**
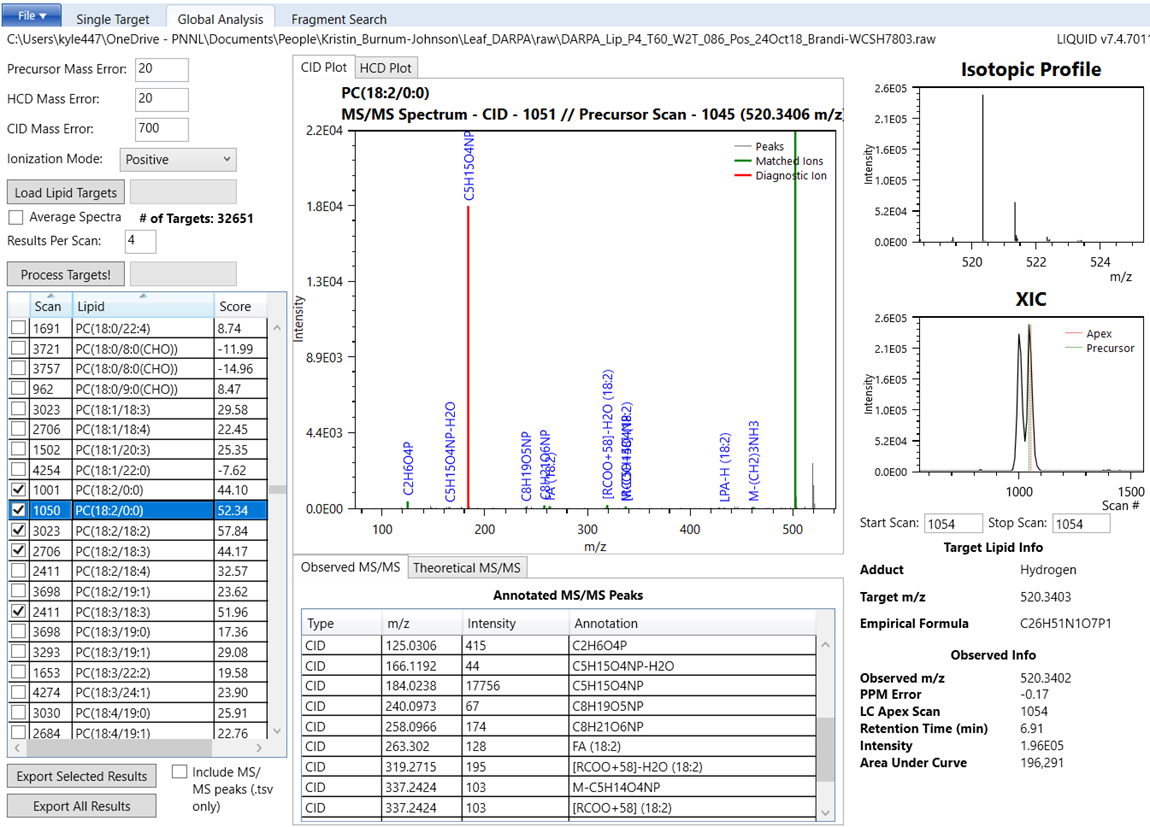
**

**
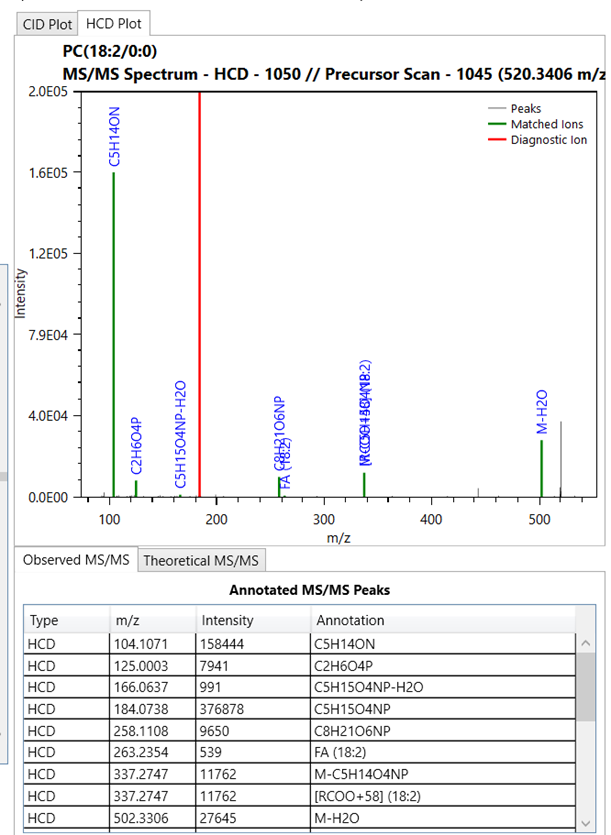
**

**PC (16:0_18:2)**

**
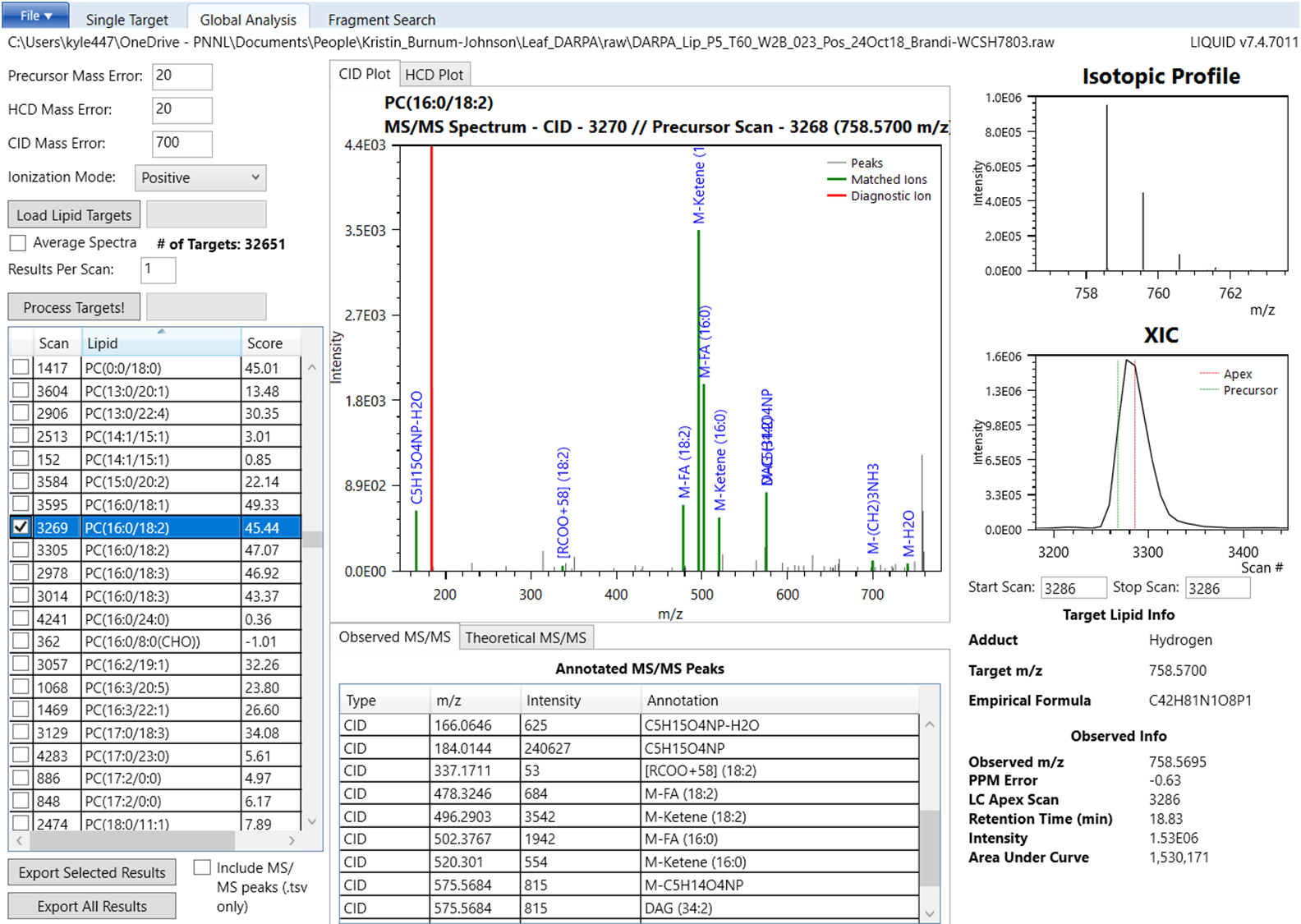
**

**PC (18:0_18:2)**

**
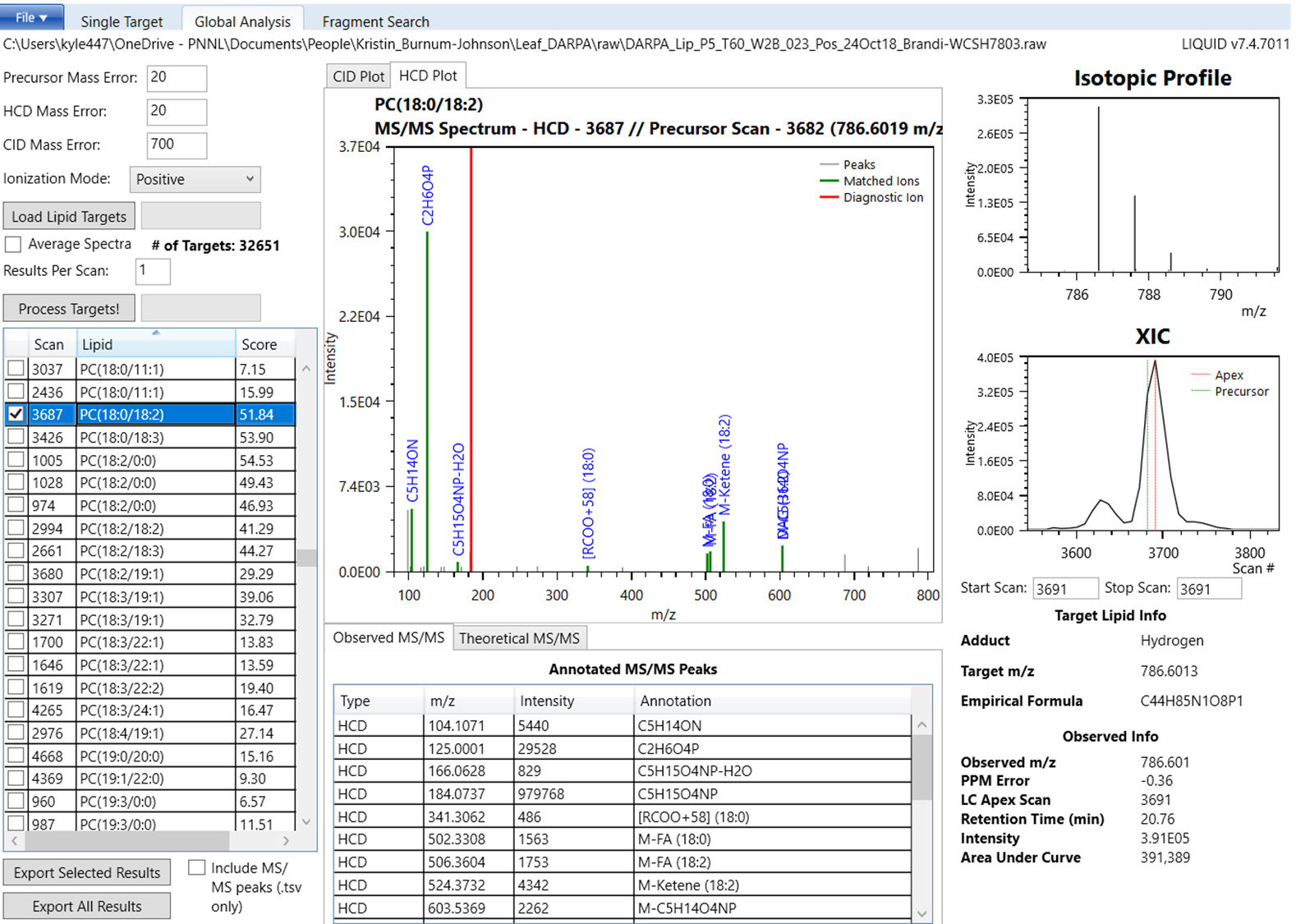
**

**PC (18:0_18:3)**

**
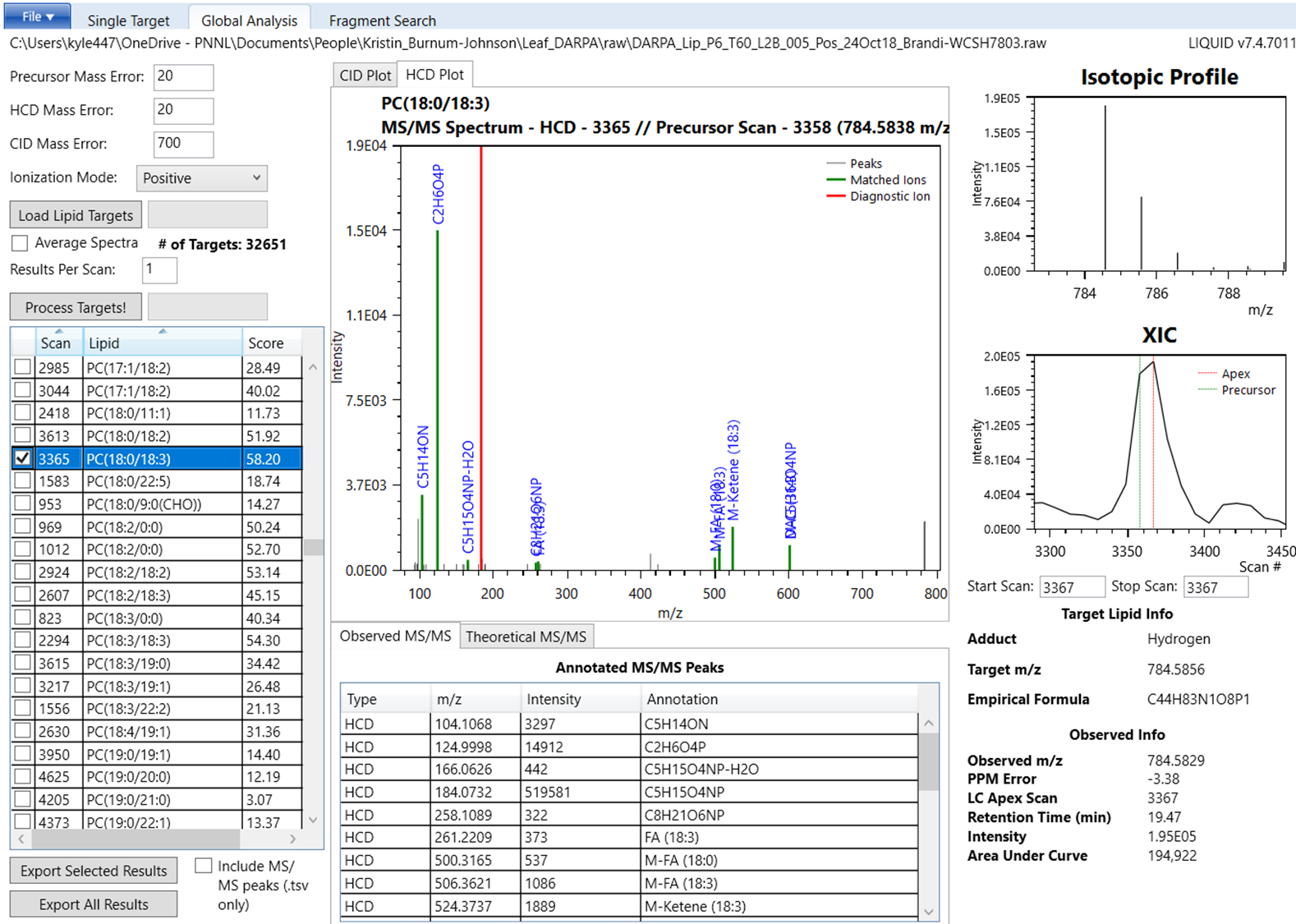
**

**PC (16:0_18:3)**

**
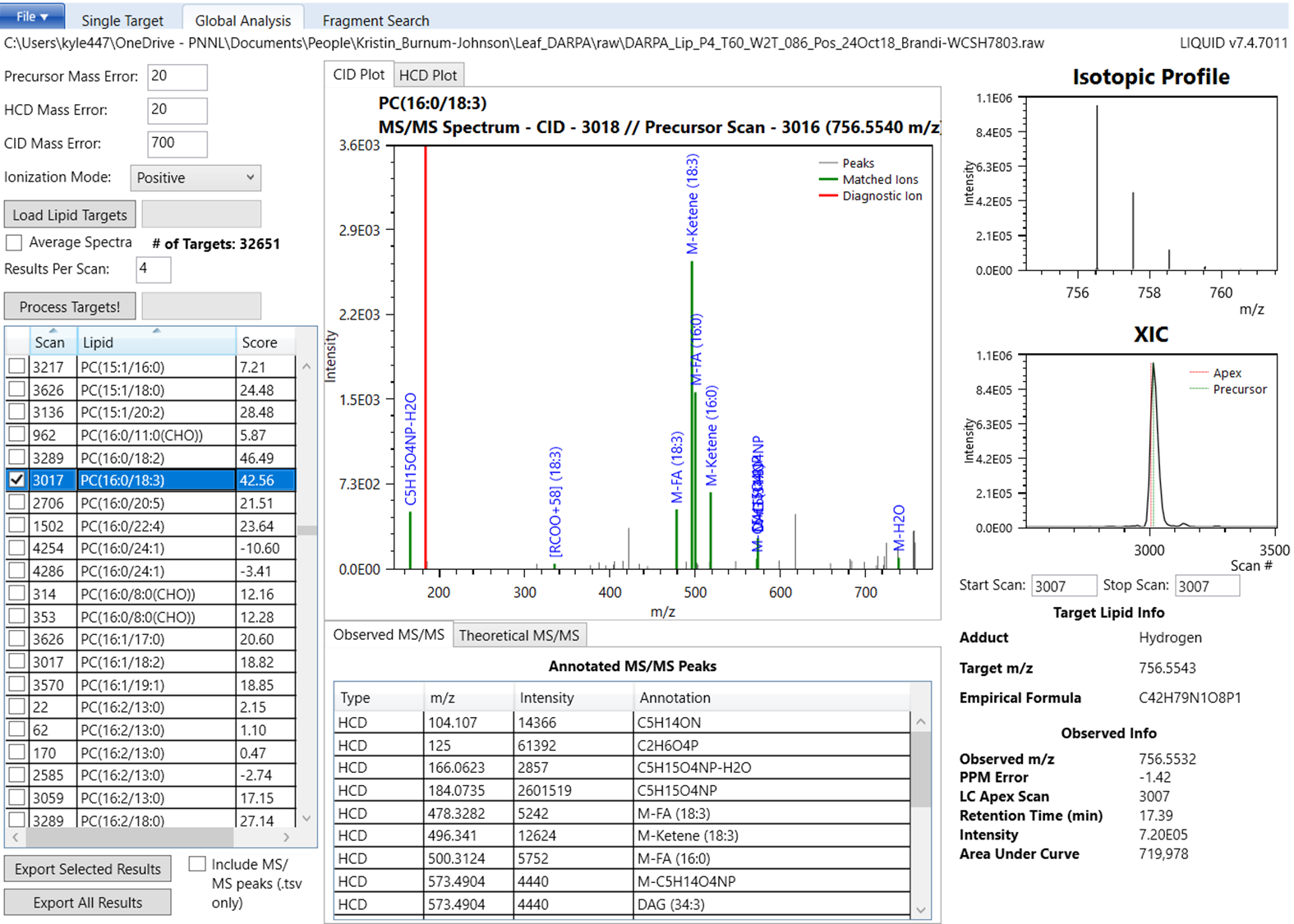
**

**PC (18:2_18:2)**

**
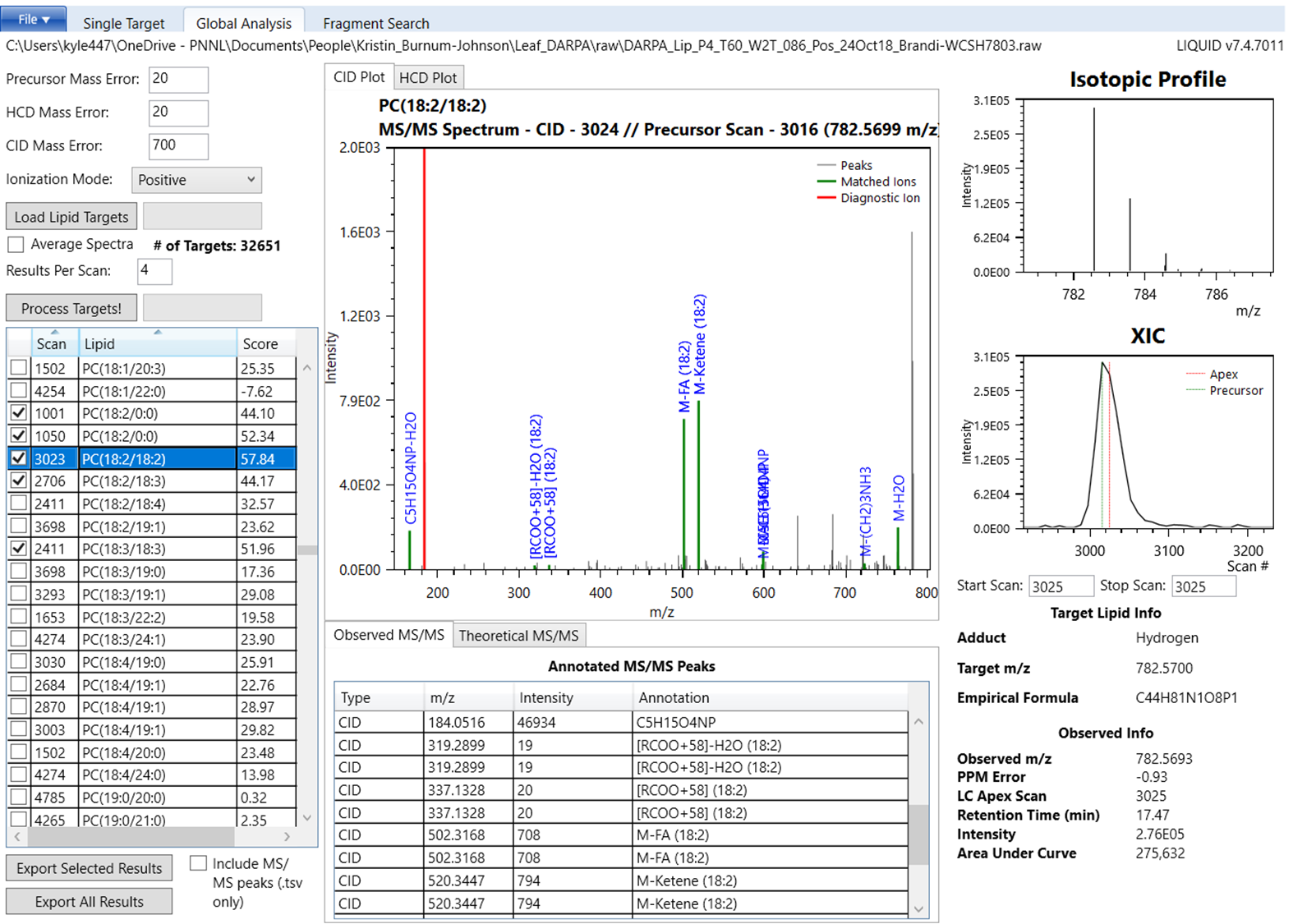
**

**PC (18:2_18:3)**

**
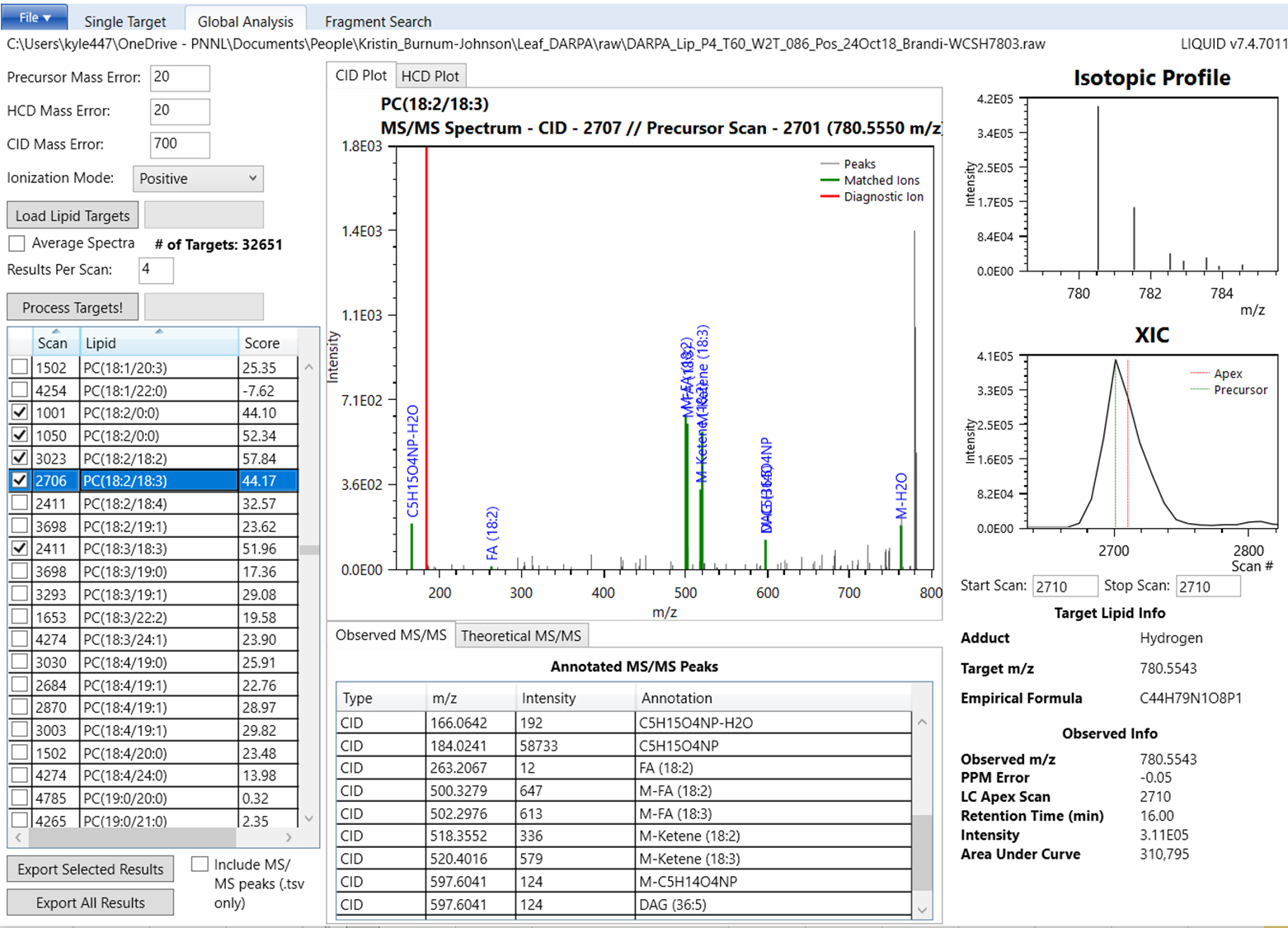
**

**PC (18:3_18:3)**

**
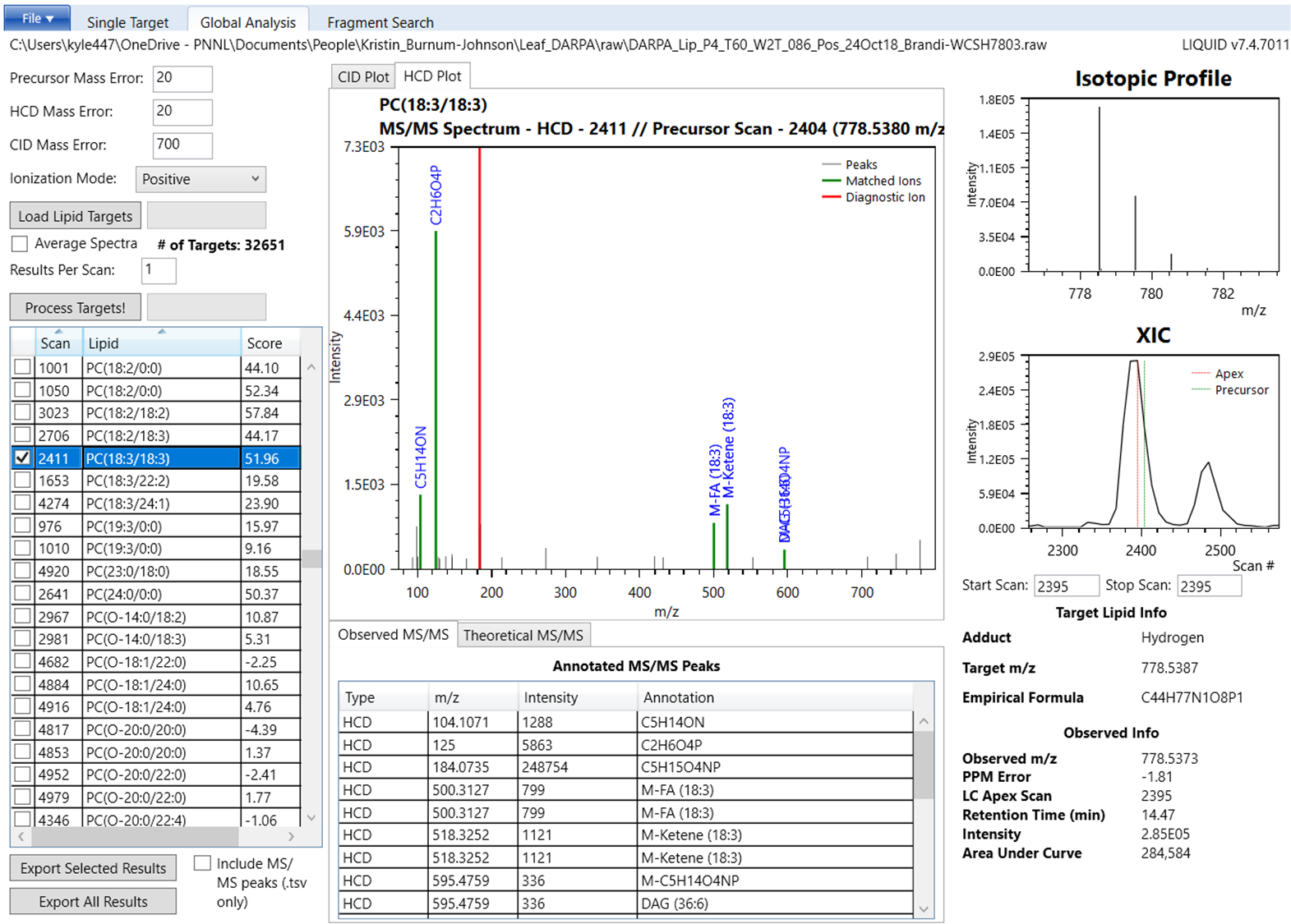
**

**SQDG (16:0_18:3)**

**
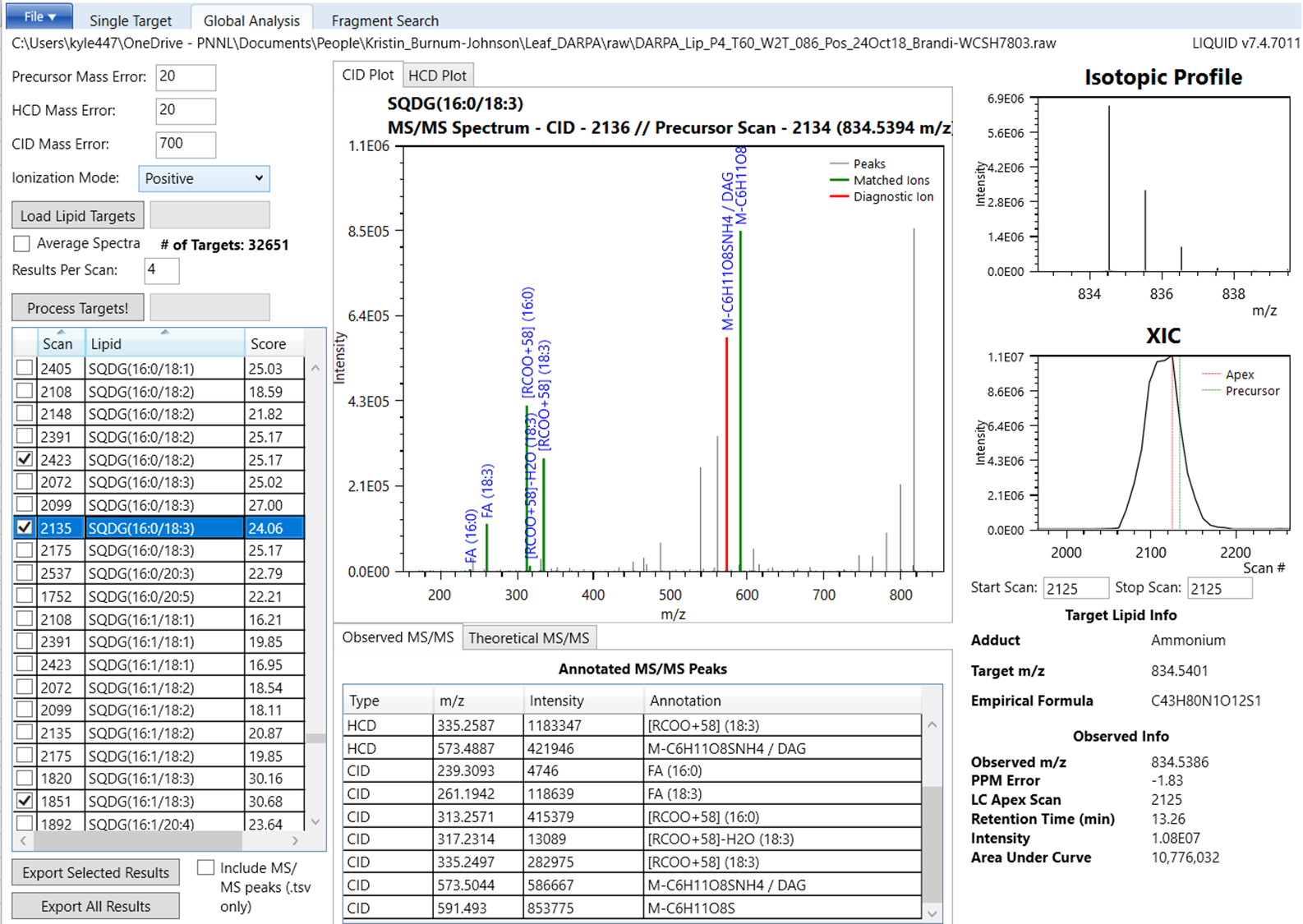
**

**LPI (16:0)**

**
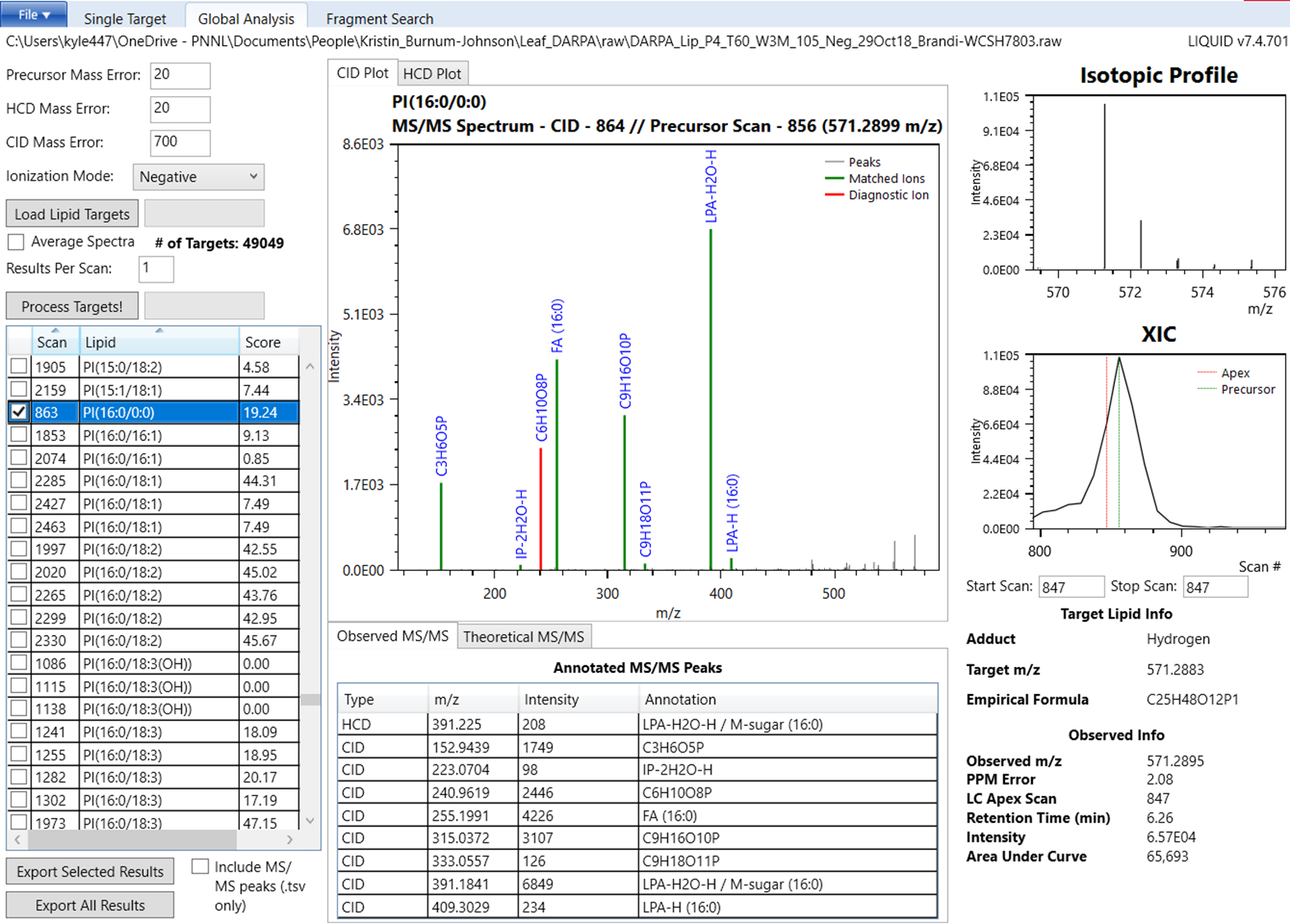
**

**PI (16:0_18:2)**

**
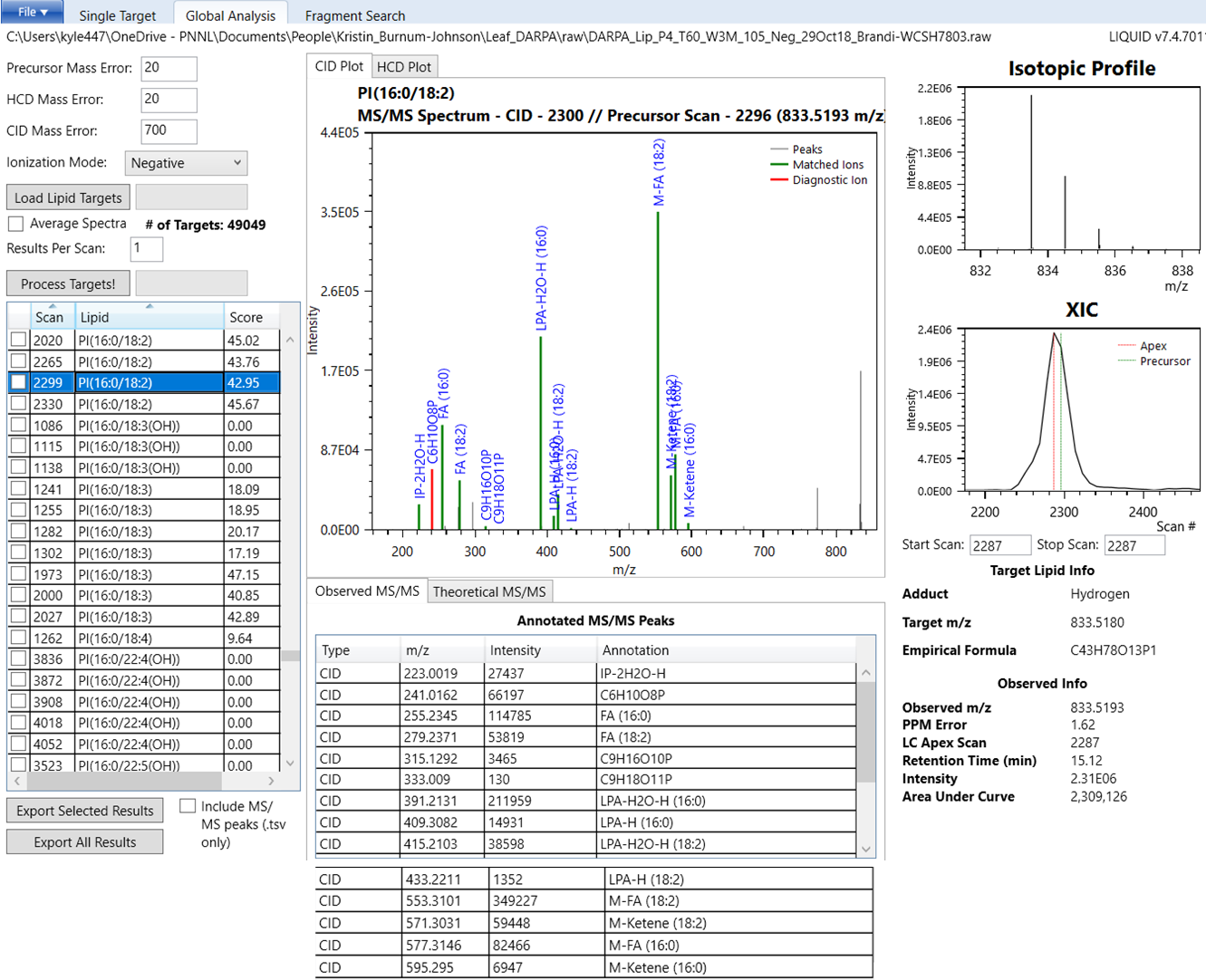
**

**PI (16:0_18:3)**

**
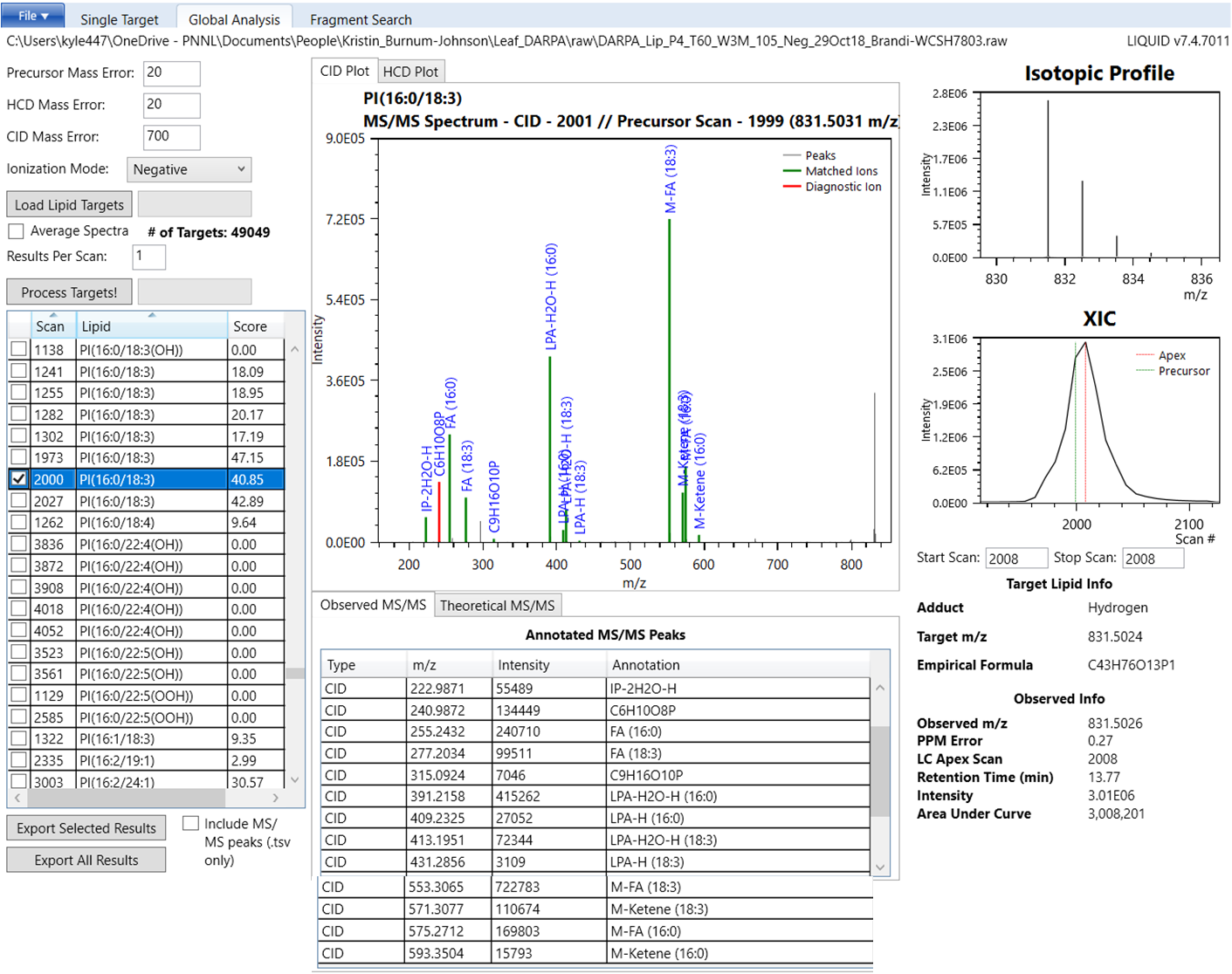
**

**References**

FOLCH, J., LEES, M. & STANLEY, G. H. S. 1957. A Simple Method for the Isolation and Purification of Total Lipides from Animal Tissues. *Journal of Biological Chemistry,* 226**,** 497-509.

KYLE, J. E., CROWELL, K. L., CASEY, C. P., FUJIMOTO, G. M., KIM, S., DAUTEL, S. E., SMITH, R. D., PAYNE, S. H. & METZ, T. O. 2017. LIQUID: an-open source software for identifying lipids in LC-MS/MS-based lipidomics data. *Bioinformatics,* 33**,** 1744-1746.

PLUSKAL, T., CASTILLO, S., VILLAR-BRIONES, A. & ORESIC, M. 2010. MZmine 2: modular framework for processing, visualizing, and analyzing mass spectrometry-based molecular profile data. *BMC Bioinformatics,* 11**,** 395.
